# Association between gestational levels of toxic metals and essential elements and cerebral palsy in children

**DOI:** 10.3389/fneur.2023.1124943

**Published:** 2023-08-17

**Authors:** Kjell Vegard F. Weyde, Adriano Winterton, Pål Surén, Guro L. Andersen, Torstein Vik, Guido Biele, Helle K. Knutsen, Cathrine Thomsen, Helle M. Meltzer, Thea S. Skogheim, Stephanie M. Engel, Heidi Aase, Gro D. Villanger

**Affiliations:** ^1^Division of Mental and Physical Health, Norwegian Institute of Public Health, Oslo, Norway; ^2^Department of Clinical and Molecular Medicine, Norwegian University of Science and Technology, Trondheim, Norway; ^3^Division of Infection Control and Environmental Health, Norwegian Institute of Public Health, Oslo, Norway; ^4^Gillings School of Global Public Health, University of North Carolina at Chapel Hill, Chapel Hill, NC, United States

**Keywords:** toxic metal, essential element, cerebral palsy (CP), pregnant women, brain develeopment, The Norwegian Mother, Father, Child Cohort Study (MoBa), Medical Birth Registry of Norway (MBRN)

## Abstract

**Introduction:**

Cerebral palsy (CP) is the most common motor disability in childhood, but its causes are only partly known. Early-life exposure to toxic metals and inadequate or excess amounts of essential elements can adversely affect brain and nervous system development. However, little is still known about these as perinatal risk factors for CP. This study aims to investigate the associations between second trimester maternal blood levels of toxic metals, essential elements, and mixtures thereof, with CP diagnoses in children.

**Methods:**

In a large, population-based prospective birth cohort (The Norwegian Mother, Father, and Child Cohort Study), children with CP diagnoses were identified through The Norwegian Patient Registry and Cerebral Palsy Registry of Norway. One hundred forty-four children with CP and 1,082 controls were included. The relationship between maternal blood concentrations of five toxic metals and six essential elements and CP diagnoses were investigated using mixture approaches: elastic net with stability selection to identify important metals/elements in the mixture in relation to CP; then logistic regressions of the selected metals/elements to estimate odds ratio (OR) of CP and two-way interactions among metals/elements and with child sex and maternal education. Finally, the joint effects of the mixtures on CP diagnoses were estimated using quantile-based g-computation analyses.

**Results:**

The essential elements manganese and copper, as well as the toxic metal Hg, were the most important in relation to CP. Elevated maternal levels of copper (OR = 1.40) and manganese (OR = 1.20) were associated with increased risk of CP, while Hg levels were, counterintuitively, inversely related to CP. Metal/element interactions that were associated with CP were observed, and that sex and maternal education influenced the relationships between metals/elements and CP. In the joint mixture approach no significant association between the mixture of metals/elements and CP (OR = 1.00, 95% CI = [0.67, 1.50]) was identified.

**Conclusion:**

Using mixture approaches, elevated levels of copper and manganese measured in maternal blood during the second trimester could be related to increased risk of CP in children. The inverse associations between maternal Hg and CP could reflect Hg as a marker of maternal fish intake and thus nutrients beneficial for foetal brain development.

## 1. Introduction

Cerebral palsy (CP) is defined as a “group of permanent disorders of the development of movement and posture” caused by a non-progressive lesion in the developing brain occurring before 2 years of age (1). Cerebral palsy is the most common motor disability in childhood, with a prevalence of about 0.2 percent (2), but affecting up to one in 10 extremely preterm born children (3). About 30% have severe gross- and fine motor impairments, being unable to walk without assistive devices or being dependent on a wheelchair and/or unable to use their hands independently. In addition, associated neurodevelopmental disorders and difficulties are common, including epilepsy, intellectual disability, eating difficulties, speech and communication difficulties as well as pain and musculoskeletal complications (4). The pathophysiology underlying CP is varied and complex, and the resulting brain injuries include brain malformations, white and grey matter injuries, though congenital malformations are identified in a minority of cases (5). CP is further classified into subtypes based on the dominating clinical symptom, i.e., spasticity, dyskinesia, or ataxia. Based upon the timing of the insult, CP is classified as postneonatal occurring from day 28 after birth to the age of 2 years, or congenital CP. Whereas the event leading to postneonatal CP is usually clearly identified, the aetiology underlying congenital CP is more obscure (6).

Genetic causes of CP are rare, but genetic factors may interact with risk factors, as the prevalence of CP is elevated among close relatives (7). Rather than being a direct cause of CP, genetic factors may interact with other risk factors, such as preterm birth, foetal growth restriction, placental dysfunction, and hypoxic ischemic insults during delivery, leading to neonatal encephalopathy and seizures (8). However, in the majority of congenital CP cases the aetiology still remains unexplained (5).

*In utero* exposure to chemicals may alone, or interacting with other factors, interfere with normal brain development leading to an early brain insult and CP. Earlier studies have found increased risk of CP diagnosis in children prenatally exposed to pharmaceuticals such as paracetamol or aspirin, as well as environmental toxicants such as pesticides and perfluoroalkyl substances (PFASs), which are known or suspected to adversely affect brain development (9–11).

Several toxic metals, such as mercury (Hg) and lead (Pb), can contribute to neurodevelopmental disorders, and neurological and motor impairments in children (12). Children and foetuses are especially vulnerable to such exposures, due to the rapid development of the brain and nervous system, lack of detoxifying enzymes and underdeveloped blood-brain barrier (13). Thus, toxic metals in maternal blood can pass the placenta and reach the foetal brain, and adversely affect foetal development of the brain and nervous system and later functioning (14–16).

In contrast to toxic (non-essential) metals, essential elements, such as copper (Cu), cobalt (Co), selenium (Se), zinc (Zn), magnesium (Mg), and manganese (Mn), are important in human physiological and biochemical processes (17). A healthy, nutrient-rich diet during pregnancy is imperative to ensure a healthy development of the foetus (18). Pregnant women are at increased risk of micro- and macronutrient deficiency due to increased demands from the foetus (19). Essential elements generally have a narrow optimal dose range, and both excessive and insufficient intake may adversely affect health (17, 20, 21).

Although there are uncertainties as to whether gestational exposure to toxic metals and essential elements are associated with risk of neurological disorders like CP in the child, there is some evidence that elevated exposure to the toxic metals Pb, Hg, arsenic (As) and the essential element Mn can impair motor function in children and adolescents (22–27). For example, symptoms of chronic Hg intoxication in childhood includes muscular hypotonia, tremor, ataxia, and coordination problems (28), and prenatal exposure to Hg has been related to poorer motor function and gross motor skills (29). A study from Japan reported a high incidence of CP following pollution of wastewater with methylmercury (MeHg) leading to high concentrations in local fish and seafood ingested by the local population, including pregnant women (30). Similar to MeHg, Pb is an established developmental neurotoxicant (31). Bansal et al. (32) found increased blood Pb concentrations in children with CP, compared to controls. However, few or no previous studies, to our knowledge, have investigated associations between gestational levels of toxic metals and essential elements, and CP diagnosis in the child.

Studies of CP diagnoses requires very large sample sizes, due to the heterogeneity and the low prevalence of CP. The present study aims to address this question by using data from a large population-based cohort, the Norwegian Mother, Father, and Child Cohort Study [MoBa; Magnus et al. (33)]. Most studies on health effects from chemical exposure have been limited to only a few exposures (34), however human populations are not exposed to only one metal at the time, but rather to a mixture of multiple metals. Metals, as well as essential elements, can act jointly (additively), or they can interact antagonistically or synergistically, yielding potentially different effects on development and health compared to when the metals/elements are considered alone (29, 35). Investigating the associations of combinations of metals/elements with health outcomes is therefore critical when it comes to research on environmental factors and children’s health (36, 37).

The aim of the present study is to investigate the associations between gestational levels of toxic metals and essential elements, individually and as mixtures, and risk of CP diagnoses in children using a prospective, population-based birth cohort.

## 2. Methods

### 2.1. Study sample

#### 2.1.1. The Norwegian Mother, Father, and Child Cohort Study (MoBa)

Participants in the current study were selected from NeuroTox, a sub-study to MoBa aimed at investigating the association of prenatal exposure to environmental toxicants and risk for neurodevelopmental and neurological disorders in children. MoBa is a population-based pregnancy cohort study conducted by the Norwegian Institute of Public Health. Participants were recruited from all over Norway during 1999–2008. The women consented to participation in 41% of the pregnancies. The cohort now includes 114,500 children, 95,200 mothers and 75,200 fathers (33). Blood samples were obtained from both parents during pregnancy and from mothers and children (umbilical cord) at birth (38). The current study is based on version 12 of the quality-assured data files. The establishment of MoBa and initial data collection was based on a license from the Norwegian Data Protection Agency and approval from The Regional Committees for Medical and Health Research Ethics. The MoBa cohort is now based on regulations related to the Norwegian Health Registry Act. The Norwegian Patient Registry (NPR) has approved the linkage between NPR and MoBa, identifying cases with a diagnosis of CP. Linkage between MoBa and the Cerebral Palsy Registry of Norway (CPRN) was also used to identify cases. The CPRN is a consent-based national medical quality register established in 2006 containing clinical data on individual children born from 1996 onwards (39). The Medical Birth Registry of Norway (MBRN) is a national health registry containing information about all births in Norway.

#### 2.1.2. Cases and controls

**Figure 1:**
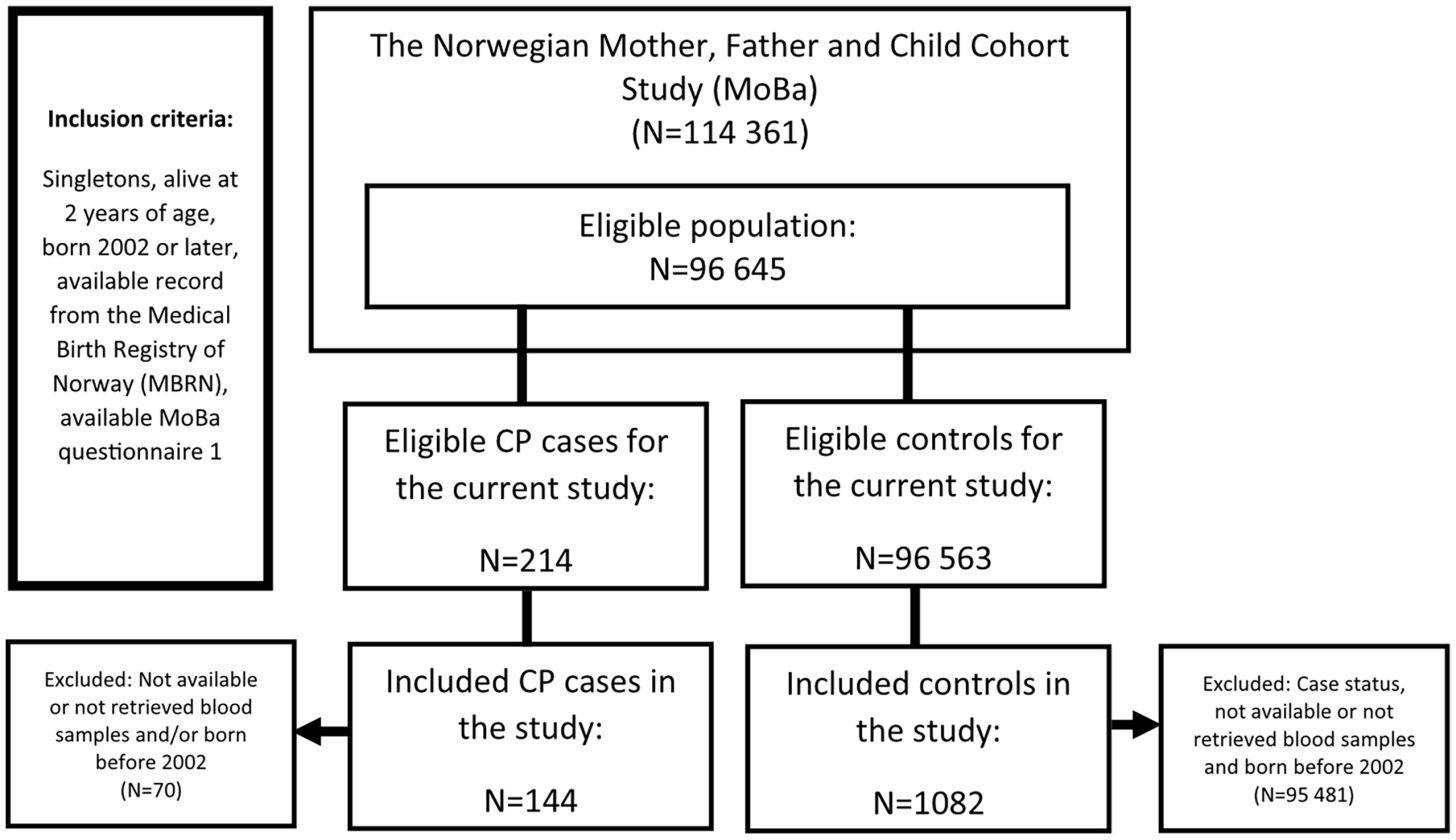
Flow chart showing the selection of cases and controls in a nested case-control study of cerebral palsy in the Norwegian Mother, Father, and Child Cohort Study (MoBa), 2002–2006.

In total, 247 MoBa children with CP diagnoses (one or more registrations of ICD-10 codes G80.0-G80.9; 40) in the CPRN (39) or the NPR (41) were identified. For children with recorded CP diagnoses in the NPR, but who were not previously captured by the CPRN (39), the diagnoses were validated according to the standard procedures of the CPRN. The inclusion criteria in the present study were (Figure 1): Singleton, alive at 2 years of age, born 2002 or later, available record from the MBRN, available maternal MoBa questionnaire 1 (gestations week 15), no registration of Downs syndrome and available maternal whole blood samples (gestations week ~18). The final sample consisted of 144 CP diagnostic cases and their mothers. Of the CP cases, 59 were categorised as hemiplegic, 43 as diplegic, 16 quadriplegic, 2 choreo-athetotic, 13 dystonic, 7 ataxic and 4 unknown or unclassifiable. The CP cases were analysed together, as there is often overlap in presentation and clinical significance between them (6).

The control population in the NeuroTox project was designed to be used for other outcomes as well, including autism spectrum disorders (ASD), attention deficit/hyperactivity disorder (ADHD), and epilepsy. This control group was randomly sampled from the eligible MoBa sample, frequency matched with birth year and child sex to all diagnostic cases in NeuroTox applying the same inclusion criteria as for the cases. Since CP, and especially ADHD and ASD, are more prevalent among boys (42), more male than female controls were sampled. The final control group consisted of 1,082 children and their mothers. The high case-control ratio leads to increased statistical power when the prevalence of cases is small (43). In addition, this study was a part of a larger studies on neurodevelopment and neurological outcomes in children, where control group was designed to fit all cases groups such as ADHD, ASD and epilepsy, where the prevalence in children is higher than that of CP (44).

The current study was approved by The Regional Committees for Medical and Health Research Ethics (ref. no. 2012/985-1). Parents enrolled in MoBa gave written consent for the use of this data.

### 2.2. Measurement of toxic metals and essential elements in maternal blood

The present study used maternal blood samples from around gestation week 18. Details about the sampling procedure and handling and storage in the MoBa biobank are described in detail elsewhere (38). Eleven toxic/non-essential metals and essential elements were determined in maternal whole blood, using inductively coupled plasma-sector field mass spectrometry (ICP-SFMS). These included the toxic metals As, cadmium (Cd), caesium (Cs), Pb and Hg, and the essential elements Co, Cu, Mg, Mn, Se, and Zn. Hg and As were measures of total Hg and total As, containing both inorganic and organic forms. In the Norwegian population, these measures will to a large degree reflect organic forms from intake of fish and seafood (45). Most samples (*n* = 1,121) were analysed at ALS laboratory group’s lab in Sweden, and some (*n* = 105) were analysed at the University of Lund (Sweden). Internal quality control samples and procedure blanks were analysed along with each batch of samples to ensure high quality of the determinations throughout the project. Additionally, reference samples were included (Seronorm Trace Elements whole blood L-1, SERO AS, Billingstad, Norway) that were used as project-specific quality control (QC) samples. Case, control, and QC samples were randomized to batch and blinded to the analysist. Details on analytical procedures, limits of detection (LOD), limits of quantification (LOQ) and quality control are presented in Supplementary materials 1 and 3. For most metals/elements, concentrations above LOQ are reported, but for As, Cd, Pb, and Hg, concentrations above LOD are reported. Metal/element concentrations are given in μg/L, except for Mg, which is given in mg/L.

Due to issues related to project design and logistics, the blood samples were pulled from the biobank and analysed for metals and elements in three separate analytical rounds (Supplementary material 1). In addition, some samples were analysed at the University of Lund in another MoBa sub-study (~round 4). To account for analytical variation across analytical rounds, the metal/element concentrations were normalised for each participant using the QC samples (Seronorm reference material) analysed in each of the analytical rounds. The approach used was similar to the scaled variation of the Ratio-G batch adjustment described in Luo et al. (46). Let *M* be the measured metal/element concentration *i* for each participant *j*. M*ij is then the analytical round adjusted metal/element concentration, and is calculated as (Eq. 1):


(1) $$M^{*}ij=Mij\times \left(\mathrm{meanQCl}/\mathrm{meanQClk}\right),$$

where meanQCl represents the geometric mean of metal/element *i* in reference samples across all analytical rounds, and meanQClk represents the geometric mean of metal/element *i* in reference samples from analytical round *k* (i.e., in the analytical round in which sample of participant *j* was measured).

### 2.3. Covariates

The covariates in the present study were obtained from three prenatal MoBa questionnaires completed in gestation weeks 15, 22, and 30 (33) and from the MBRN. The following covariates were considered: from MoBa: maternal education (up to and including 4 years of university/college vs. 5 years of university/college or more), maternal smoking during pregnancy (daily/sometimes vs. no), maternal seafood consumption during pregnancy obtained from the food frequency questionnaire (gestation week 22), and maternal pre-pregnancy body mass index; from MBRN: child sex, child birth year (2002–2005 vs. 2006–2009), parity (0 vs. 1^+^), maternal age at delivery (in years), gestational age, and birth weight. A minimal adjustment set was identified using directed acyclic graphs made at dagitty.net [DAGs; Textor et al. (47); see Supplementary material 11 and Table 1], and included maternal education, maternal seafood intake during pregnancy, maternal age at delivery, maternal smoking during pregnancy, and parity. Sex and birth year were additionally included as covariates, since these variables are important in relation to CP and metal/element exposure, respectively.

**Table 1:** Descriptive information for controls, cases, and the total study sample in a nested case-control study of cerebral palsy in the Norwegian Mother, Father, and Child Cohort Study (MoBa), 2002–2006.

	Controls	CP cases	Total
	*N* = 1,082	*N* = 144	*N* = 1,226
Maternal age [mean (SD)]^a^	30.0 (4.5)	30.4 (4.7)	30.1 (4.5)
Seafood intake, pregnancy [mean (SD)]^a^	36.5 (21.8)	33.7 (21.2)	36.2 (21.8)
Missing	128	15	143
Maternal folate intake, pregnancy [mean (SD)]^b^	511.2 (272.3)	547.5 (277.8)	515.5 (273.1)
Missing	242	31	273
Gestational age, days [mean (SD)]^b^	279.6 (11.7)	265.0 (31.3)	277.9 (16.0)
Missing	4	1	5
*Parity* ^a^			
0	459 (42.4%)	80 (55.6%)	539 (44.0%)
1^+^	623 (57.6%)	64 (44.4%)	687 (56.0%)
*Maternal education* ^a^			
<5 year university	357 (33.8%)	49 (34.8%)	406 (33.9%)
≥5 year university	698 (66.2%)	92 (65.2%)	790 (66.1%)
Missing	27	3	30
*Maternal smoking, pregnancy* ^a^			
No	937 (86.6%)	132 (91.7%)	1,069 (87.2%)
Yes	145 (13.4%)	12 (8.3%)	157 (12.8%)
Maternal pre-pregnancy BMI^b^	23.4 (5.9)	24.5 (7.1)	23.5 (6.1)
*Birth year* ^a^			
<2006	867 (80.1%)	65 (45.1%)	932 (76.0%)
≥2006	215 (19.9%)	79 (54.9%)	294 (24.0%)
*Sex* ^a^			
Boys	744 (68.8%)	83 (57.6%)	827 (67.5%)
Girls	338 (31.2%)	61 (42.4%)	399 (32.5%)

a Adjustment variables in the analyses.

b Not part of minimal adjustment set.

### 2.4. Statistical analyses

Preliminary analyses (see Supplementary material 12) indicated that some metal/element outliers could influence the estimates of the individual metal/element-CP relationships. To deal with outliers, a winsorization approach was used (48) where metal/element concentrations below the first and above the 99^th^ percentiles were replaced with the value of the first and 99th percentile, respectively. Then all metal/element concentrations were natural log transformed to reduce skewness.

Multiple imputation (*M* = 20) was used to replace missing values with Amelia II in R (49). As, Cd and Co had some missing values due to concentrations below LOD or LOQ. Therefore, lower (≈0) and upper (LOQ for Cd and Co; LOD for As) bounds were specified for these variables in the imputation. Missing Mg and Cs (not analysed at the University of Lund) were imputed based on their log-normal distributions. We also imputed missing covariates (see Tables 1, 2). Imputations were based on the following variables: CP diagnoses, log-transformed metal/element concentrations, maternal age, maternal smoking during pregnancy, parity, maternal education, child sex, maternal pre-pregnancy BMI, maternal seafood intake during pregnancy, birth year, gestational age in days, and total maternal folate intake during pregnancy. Kernel density plots were used to confirm that the imputed values seemed reasonable.

**Table 2:** Batch adjusted metal concentrations (μg/L or mg/L) for controls, cases, and the total study sample in a nested case-control study of cerebral palsy in The Norwegian Mother, Father, and Child Cohort Study (MoBa), 2002–2006.

	Controls						CP cases						Total					
	*N* = 1,082						*N* = 144						*N* = 1,226					
	Mean (SD)	Min	10%	50%	90%	Max	Mean (SD)	Min	10%	50%	90%	Max	Mean (SD)	Min	10%	50%	90%	Max
Hg (ug/L), adjusted	1.4 (0.9)	0.1	0.6	1.2	2.5	8.4	1.2 (0.7)	0.1	0.3	1.0	2.2	3.5	1.4 (0.9)	0.1	0.5	1.1	2.4	8.4
As (ug/L), adjusted	2.4 (3.1)	0.1	0.6	1.6	4.8	51.9	2.1 (2.3)	0.2	0.8	1.4	3.7	14.9	2.4 (3.0)	0.1	0.6	1.6	4.8	51.9
Missing^a^	12						0						12					
Cd (ug/L), adjusted	0.3 (0.3)	0.0	0.1	0.2	0.5	3.0	0.2 (0.2)	0.0	0.1	0.2	0.3	2.1	0.2 (0.3)	0.0	0.1	0.2	0.5	3.0
Missing^a^	22						0						22					
Pb (ug/L), adjusted	10.0 (5.5)	2.0	5.6	9.0	15.1	85.8	9.8 (7.6)	1.0	4.9	8.7	14.4	86.0	10.0 (5.8)	1.0	5.5	8.9	15.0	86.0
Mn (ug/L), adjusted	11.2 (8.8)	3.4	6.6	9.8	15.2	162.7	13.4 (12.9)	1.9	6.7	10.2	19.4	105.1	11.5 (9.4)	1.9	6.6	9.8	15.5	162.7
Se (ug/L), adjusted	92.3 (20.4)	45.8	71.6	89.7	116	303.5	91.9 (18.7)	56.7	69.3	90.0	121.7	144.1	92.3 (20.2)	45.8	71.1	89.7	117.0	303.5
Co (ug/L), adjusted	0.3 (0.9)	0.0	0.1	0.2	0.4	29.3	0.2 (0.2)	0.0	0.1	0.2	0.3	1.4	0.3 (0.9)	0.0	0.1	0.2	0.4	29.3
Missing^a^	32						1						33					
Cs (ug/L), adjusted	2.4 (0.9)	0.9	1.5	2.3	3.4	8.4	2.3 (0.8)	0.8	1.4	2.2	3.3	5.3	2.4 (0.9)	0.8	1.5	2.2	3.3	8.4
Missing^a^	103						2						105					
Cu (ug/L), adjusted	1,583 (243)	778	1,300	1,551	1891	3,178	1,623 (282)	939	1,277	1,609	1980	3,069	1,588 (248)	778	1,297	1,554	1903	3,178
Zn (ug/L), adjusted	5,491 (1088)	1,641	4,090	5,460	6,837	10,294	5,203 (909)	1,432	4,056	5,240	6,264	7,433	5,457 (1072)	1,432	4,086	5,436	6,751	10,294
Mg (mg/L), adjusted	30.3 (3.5)	18.3	25.9	30.3	34.8	45.0												

a Missing was due to values below level of detection.

Correlations in one of the imputed datasets among the measured toxic metals and essential elements in maternal blood were investigated using Spearman correlation.

All regression models are based on multiple imputed data, unless otherwise mentioned, and adjusted for child sex, birth year, parity, maternal education, maternal smoking during pregnancy, maternal age at delivery, and maternal seafood consumption during pregnancy.

#### 2.4.1. Identifying important metals/elements in the association with CP

Simultaneously including multiple correlated exposure variables can produce unstable estimates and inflated standard errors when running traditional regression models (50). To overcome this limitation, a method for regularization and variable selection was used: elastic net regression (51) (see Supplementary material 2), to identify metal/element exposures important for CP. The covariates were not penalized. Elastic net regression is a suitable method to identify the most important elements in relation to the outcome within a mixture, which then can be used to characterise in the independent exposure-response relationships of the selected mixture member(s) (50).

To ensure the robustness of the elastic net results, stability selection was performed. In stability selection, variables that are only weakly related to the outcome are more likely to be filtered out, due to more noise being introduced into the data (52). In short, random sampling from the original data with replacement was done 200 times, yielding 200 new datasets. In each of these datasets, 20 multiple imputed (MI) datasets were made. Elastic net was run in every MI dataset, and it was calculated how often, on average, the exposures were selected. Thus, each randomly drawn dataset yielded selection probability estimate for each exposure. The mean of the selection probabilities across the 200 randomly drawn datasets was then calculated. A permutation procedure was used to calculate *p*-values from the elastic net regression with stability selection.

The selection probabilities and *p*-values were in combination used as an indication of the strength of the association between exposure and outcome. Exposures with *p*-value ≤0.05 (and a high selection probability; >0.6) were selected for further analyses and entered into multivariable, adjusted logistic regression models (co-adjusted for other selected exposures) in order to obtain odds ratios (ORs) for CP. The regression model was run in each imputed dataset, and resulting ORs (no CIs or *p*-values were considered) were combined using Rubin’s rules (53). For reason of comparison with the variable selection results, we ran multivariable adjusted linear regression models with all individual metal/elements. Estimates are given as OR per interquartile increase in metal/element.

Whether the associations between selected individual exposures and risk for CP deviated from a monotonic dose-relationship was further investigated using multivariable adjusted natural splines with knots at 10th, 50th, and 90th percentiles. A model with the exposure as linear term was then tested against a model with the exposure modelled as splines using likelihood ratio (lr) tests.

#### 2.4.2. Identifying important two-way interactions

In order to detect possible multiplicative two-way interactions between individual metals/elements, we performed the elastic net stability selection described above. Previous studies have found associations with CP for sex and parental education (42, 54, 55). Thus, we also investigated interaction (effect measure modification) by child sex and maternal education (as proxy for SES). All independent variables were standardized. Pairwise interaction terms between all the metals/elements and between metals/elements and sex and maternal education were generated. The selected interaction terms (with *p*-value ≤0.1 and high selection probability) were included in a logistic regression model and visualized using line graphs.

#### 2.4.3. Assessment of total metal/element mixture effect using quantile *g*-computation

The effect of individual exposures may be small and difficult to identify, while the joint effect of multiple chemicals in the mixture can cause stronger effects than that of single exposures (35). Therefore, analyses to investigate the joint effects of three mixtures were performed using three models: (1) a mix containing all 11 metals/elements (MixAll), (2) a mix containing five toxic metals (As, Hg, Cd, Cs and Pb; MixTox), and (3) a mixture containing six essential elements (Mn, Cu, Co, Se, Mg and Zn; MixEssential). For this purpose, quantile *g*-computation (qgcomp) analyses with the R package qgcomp (56) were used. In this approach the exposure variables are used to construct a weighted exposure index of the mixture, reducing dimensionality and possible multicollinearity problems. The index is included in a regression model along with covariates of interest, yielding an overall effect estimate for the mixture. A one-unit increase in the mixture corresponds to all metals/elements in the mixture increasing by one unit. Weights are constructed to represent the relative strength of each exposure in relation to the outcome. The model was run in each imputed dataset, with exposures categorized as quartiles. The estimates with 95% CIs were combined using Rubin’s rules (53). Additionally, the interactions identified in the elastic net analysis were tested, along with potential non-linear effects of the mixture using the qgcomp.boot function.

#### 2.4.4. Sensitivity analyses

Sensitivity analyses were performed in regression models of selected metals only. Analyses were stratified by children of mothers with folate intake during pregnancy below (*N* = 613) or above (*N* = 613) the median. Analyses were restricted to children born to term (born in gestational week 37 or later; *N* = 1,138), children not born small for gestational age [SGA; Maršál et al. (57); *N* = 1,199], and children of mothers who did not smoke during pregnancy (*N* = 1,069). The main analysis was also done without winsorizing (*N* = 1,226) and on complete cases (subjects without missing values; *N* = 1,002). In addition, due to multiple comparison, the results from the elastic net stability selection were assessed controlling the false discovery rate (fdr) at *α* = 0.05 (*α* = 0.1 for the interactions), according to a method proposed by Ahmed et al. (58) (see Supplementary material 2 for details).

All analyses were done in R version 4.0 (59), using the packages *glmnet* (60), *qgcomp* (56), *forestplot* (61), *ggplot2* (62), *sjPlot* (63), *ggExtra* (64), *ggeffects* (63), *stargazer* (65), *mitools* (66), *Amelia II* (49), *reshape* (67), *and splines* (68). Example scripts for the analysis are available at https://osf.io/5867z/. Data from MoBa and MBRN used in this study are managed by Norwegian Institute of Public Health and can be made available to researchers, provided approval from the Regional Committees for Medical and Health Research Ethics (REC), compliance with the EU General Data Protection Regulation (GDPR) and approval from the data owners. The consent given by the participants does not open for storage of data on an individual level in repositories or journals. Researchers who want access to data sets for replication should apply through helsedata.no. Access to data sets requires approval from The Regional Committee for Medical and Health Research Ethics in Norway and an agreement with MoBa.

## 3. Results

The characteristics of the study population is presented in Table 1. Compared to mother of controls, mothers of children with CP had a lower seafood consumption during pregnancy and had slightly higher pre-pregnancy BMI. A larger proportion of children with CP were first-borns, were girls and had lower birth weight and gestational age than compared to the controls (Table 1), which is in line with previous literature (69, 70). Table 2 and Supplementary material 4 present the distribution of adjusted and non-adjusted (original) gestational concentrations of metals/elements, respectively.

**Figure 2:**
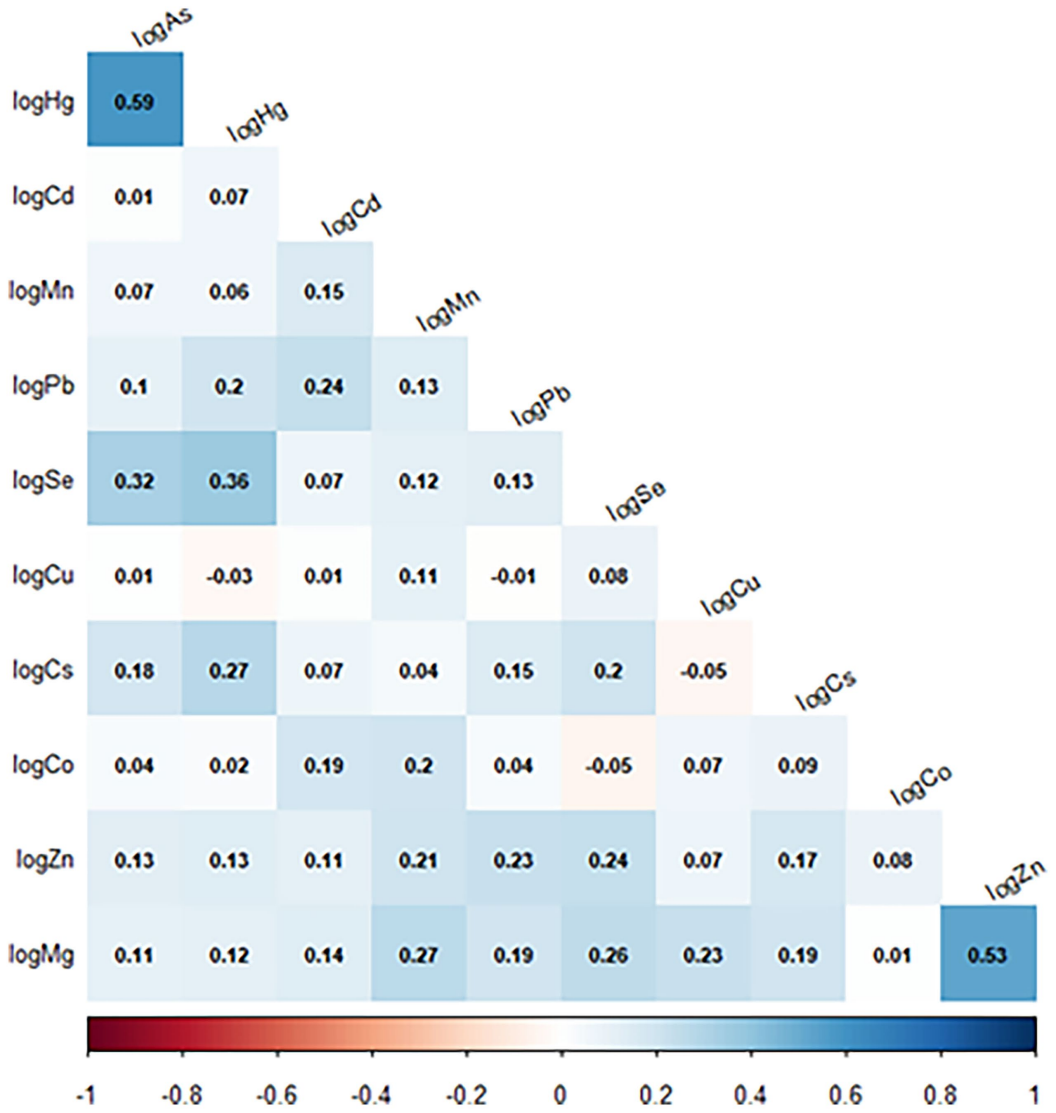
Spearman correlation between the metals/elements (adjusted concentrations) in a nested case-control study of cerebral palsy in the Norwegian Mother, Father, and Child Cohort Study (MoBa), 2002–2006. *N* = 1,226. Arsenic (As); cadmium (Cd); cesium (Cs); cobalt (Co); copper (Cu); lead (Pb); magnesium (Mg); manganese (Mn); mercury (Hg); selenium (Se); zinc (Zn).

Spearman correlations between the log-transformed concentrations of the metals and elements showed low to moderate correlations (Figure 2), with the highest correlations being those between As and Hg (*r* = 0.59) and Mg and Zn (*r* = 0.53).

**Figure 3:**
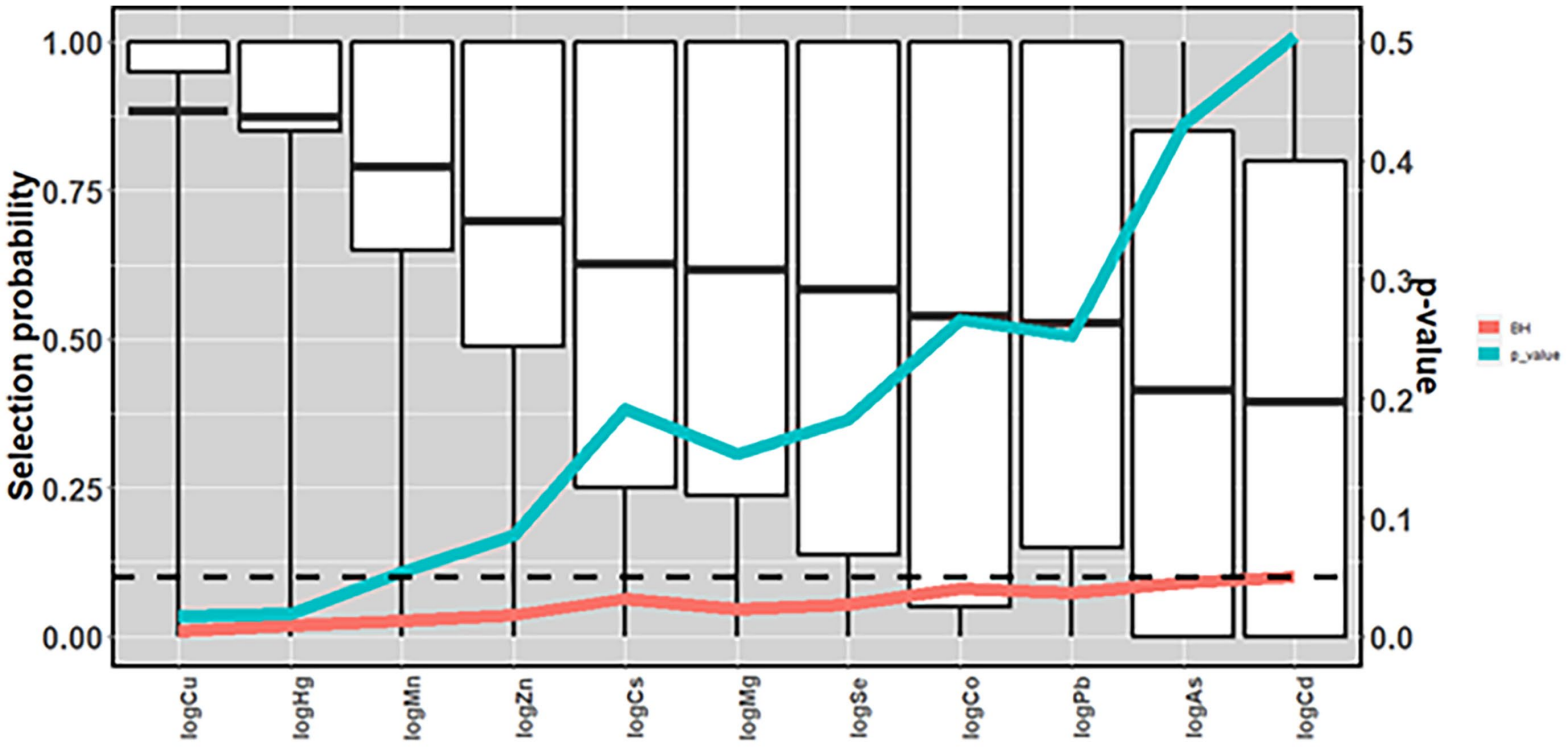
Mean selection probability (boxplot) in a nested case-control study of cerebral palsy in the Norwegian Mother, Father, and Child Cohort Study (MoBa), 2002–2006. *N* = 1,226. Based on elastic net regression in 2000 datasets (20 multiple imputed datasets in each of 100 randomly drawn datasets with replacement), and calculated *p*-values [based on 240,000 elastic net runs (once in each of 10 multiple imputed datasets in each of 20 randomly sampled datasets with replacements in each of 1,200 permuted datasets)], and Benjamini and Hochberg false discovery rate thresholds. All analyses adjusted for maternal age, parity, maternal smoking during pregnancy, maternal education, sex, and birth year. Arsenic (As); Benjamini and Hochberg false discovery rate thresholds (BH); cadmium (Cd); cesium (Cs); cobalt (Co); copper (Cu); lead (Pb); magnesium (Mg); manganese (Mn); mercury (Hg); selenium (Se); zinc (Zn).

**Figure 4:**
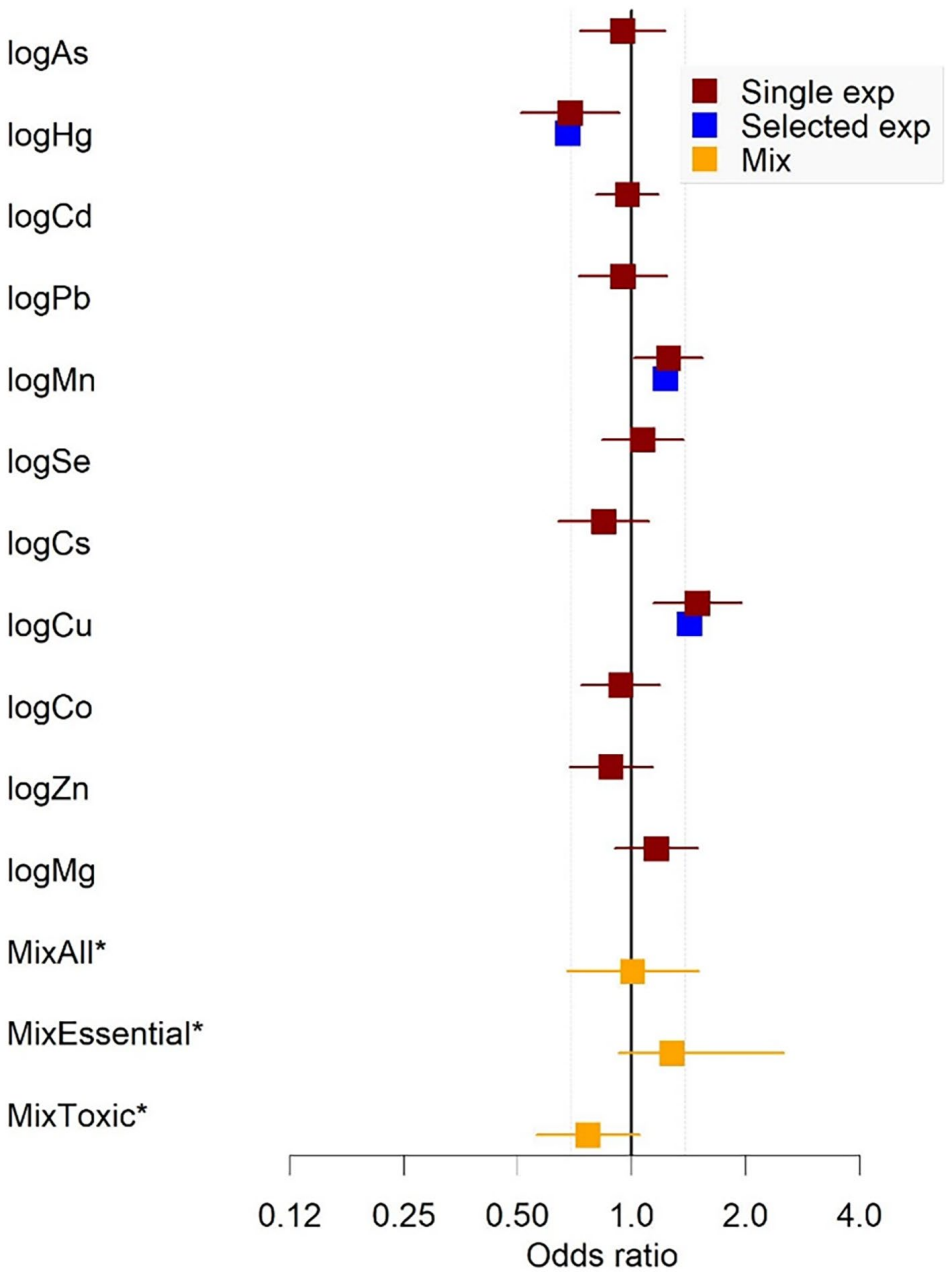
Estimates from logistic regression models of single exposures (with 95% CI), selected exposure (co-adjusted) and joint mixture exposure in a nested case-control study of cerebral palsy in the Norwegian Mother, Father, and Child Cohort Study (MoBa), 2002–2006. *N* = 1,226. Odds ratios for single metals/elements are per interquartile range increase in exposure. Yellow squares are estimates from the quantile *g*-computation analyses and represent ORs per one quartile increase in mix. ^*^MixAll = all 11 metals/elements; MixEssential = Co, Cu, Mn, Se, Mg, Zn; MixToxic = As, Hg, Cd, Pb, Cs. All models were fit in 20 multiply imputed datasets, adjusting for maternal age, parity, maternal smoking during pregnancy, maternal education, sex, and birth year. The estimates were combined using Rubin’s rules. Arsenic (As); cadmium (Cd); cesium (Cs); cobalt (Co); copper (Cu); lead (Pb); magnesium (Mg); manganese (Mn); mercury (Hg); selenium (Se); zinc (Zn).

Using elastic net models in conjunction with stability selection and the permutation approach, maternal levels of Cu (*p* = 0.018, *P*_sel_ = 0.88), Hg (*p* = 0.019, *P*_sel_ = 0.87) and Mn (*p* = 0.055, *P*_sel_ = 0.79); had the strongest associations with odds of CP in children. These associations did not remain when comparing with *p*-values adjusted for multiple comparisons (*p* > *p*_FDR_; Figure 3 and Supplementary material 5). When Mn, Hg and Cu were included in the same multivariable adjusted, logistic regression models, this resulting in the following effect estimates (ORs; per interquartile range increase in exposure): Higher maternal levels of Cu (OR = 1.40) and Mn (OR = 1.2) were associated with an increased risk of CP in the child, whereas Hg was associated with a lowered risk (OR = 0.68) (Figure 4 and Supplementary material 6). A similar pattern was observed in multivariable adjusted logistic regression models of single exposures: Hg [OR = 0.69, 95% CI = (0.51, 0.92)], Mn [OR = 1.30, 95% CI = (1.00, 1.50)] and Cu [OR = 1.50, 95% CI = (1.10, 2.00)] (Figure 4 and Supplementary material 6).

Modelling the relationship between the selected metals/element exposures and CP as natural splines with knots at 10th, 50th, and 90th percentiles, indicated no departure from linearity in the relationship between prenatal levels of Cu, Mn and Hg and odds of CP in the child (Supplementary material 6).

Restricting the analyses to children of non-smokers, children born at term, non-SGA children, or complete cases only, the effect estimates remained relatively unaffected (Supplementary material 14 and Supplementary material 7). When stratified by median maternal folate intake during pregnancy, the results in the lower intake group attenuated somewhat, whereas the higher intake group tended to be further away from one.

**Figure 5:**
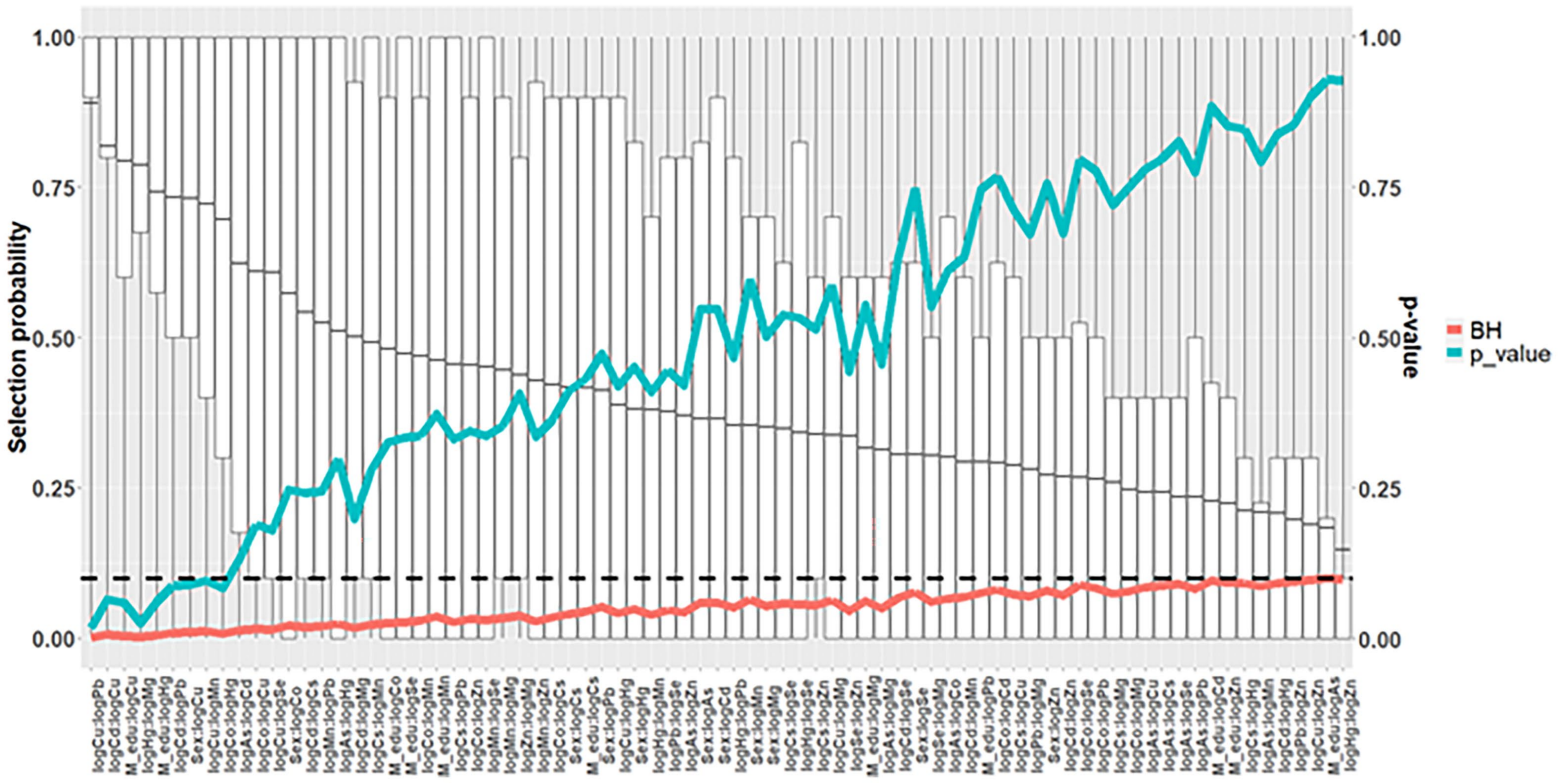
Mean selection probability (boxplot) for two-way interaction terms in a nested case-control study of cerebral palsy in the Norwegian Mother, Father, and Child Cohort Study (MoBa), 2002–2006. *N* = 1,226. Based on elastic net regression in 4000 datasets (20 multiple imputed datasets in each of 200 randomly drawn datasets with replacement; alpha = 0.9), and calculated *p*-values [based on 1,000,000 elastic net runs (once in each of 5 multiple imputed datasets in each of 20 randomly sampled datasets with replacements in each of 10,000 permuted datasets)], and Benjamini and Hochberg false discovery rate thresholds. All analyses adjusted for maternal age, parity, maternal smoking during pregnancy, maternal education, sex, and birth year. Arsenic (As); Benjamini and Hochberg false discovery rate thresholds (BH); cadmium (Cd); cesium (Cs); cobalt (Co); copper (Cu); lead (Pb); magnesium (Mg); manganese (Mn); mercury (Hg); selenium (Se); zinc (Zn).

**Figure 6:**
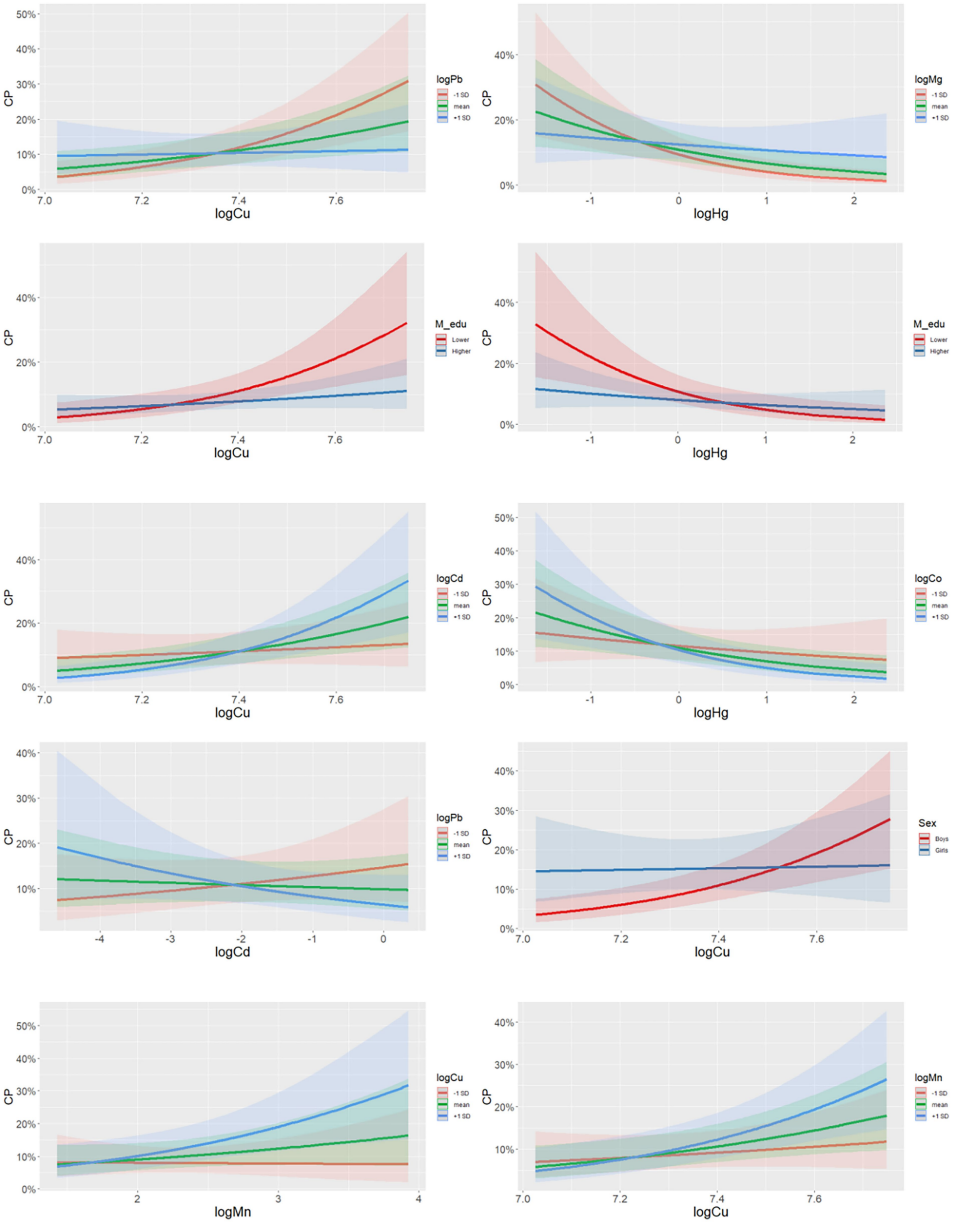
Graphs of selected two-way interaction terms in a nested case-control study of cerebral palsy in the Norwegian Mother, Father, and Child Cohort Study (MoBa), 2002–2006. *N* = 1,226. Based on a single imputed dataset. All analyses adjusted for maternal age, parity, maternal smoking during pregnancy, maternal education, sex, and birth year. *N* = 1,226. Arsenic (As); cadmium (Cd); cerebral palsy (CP); cesium (Cs); cobalt (Co); copper (Cu); lead (Pb); magnesium (Mg); manganese (Mn); mercury (Hg); selenium (Se); zinc (Zn).

Several two-way interactions were identified: Cu*Pb (*p* = 0.017, *P*_sel_ = 0.89;), Cd*Cu (*p* = 0.065, *P*_sel_ = 0.82), Maternal education*Cu (*p* = 0.059, *P*_sel_ = 0.79), Hg*Mg (*p* = 0.025, *P*_sel_ = 0.79), Maternal education*Hg (*p* = 0.061, *P*_sel_ = 0.74;), Cd*Pb (*p* = 0.088, *P*_sel_ = 0.73), Child sex*Cu (*p* = 0.090; *p*_FDR_ = 0.010), Cu*Mn (*p* = 0.096, *P*_sel_ = 0.72), and Co*Hg (*p* = 0.084, *P*_sel_ = 0.70) (Figure 5 and Supplementary material 8). None of the relationships remained when controlling for multiple comparisons (*p* > *p*_FDR_; Figure 5 and Supplementary material 6). The identified interaction terms (i.e., *p* ≤ 0.10) are visualized using stratified, linear regression plots in Figure 6. The plots show, for example, that the positive relationship for Cu with CP was larger for lower Pb levels and higher levels of Cd, and for children of less educated mothers, and for boys. The relationship between Mn and CP was stronger for higher prenatal levels of Cu. The inverse relationship for Hg was largest in children of less educated mothers, and for lower levels of Mg.

Using qgcomp, there was no significant association between the metal/element mixture [MixAll—OR = 1.00, 95% CI = (0.67, 1.50), Supplementary material 6]. When restricting the analysis to the toxic metals mixture (MixTox) there was an inverse association with CP [OR = 0.77, 95% CI = (0.56, 1.00); Figure 4 and Supplementary material 6], with Hg being the largest contributor to this association (Supplementary material 13). The association did not remain after excluding Hg [OR = 0.83, 95% CI = (0.61, 1.12)], or when comparing with false discovery rate for multiple comparisons (Supplementary material 6). No significant effect was found for the essential elements mixture [MixEssential—OR = 1.30, 95% CI = (0.93, 2.50), Supplementary material 6]. The MixAll model including all the interactions identified using elastic net regression did not find significant effects [OR = −0.05, 95% CI = (−0.3, 0.27)], nor did the model accounting for nonlinear effects [quadratic fit OR = 0.004, 95% CI = (−0.008, 0.008)].

## 4. Discussion

The present study is among the first to investigate the associations between multiple toxic metals and essential elements and their mixtures measured in maternal blood during pregnancy and risk of CP diagnosis in the child within a population-based birth cohort. As a first mixture approach, a penalized regression analysis was used to identify potentially important elements in the mixture in relation to risk of CP. Mn, Cu, and Hg appeared the most important. Follow-up logistic regression models of these individual elements indicated an increased risk of CP in the child associated with increased maternal levels Cu and Mn. In addition, maternal level of Hg was inversely related to CP. Using a similar approach, several two-way interactions were identified among metals/elements that appeared important for risk of CP, as well as effect measure modification by child sex and SES. The second mixture approach, investigating the joint effect of the mixture(s), revealed no significant effect for the metal/element mixture, but the mixture containing toxic metals revealed an inverse association with CP, in which Hg appeared to have the most influential, though it must be noted that since it will be missing co-confounding or co-exposure effects, it might not be very reliable. The discrepancy between the effects of the overall (MixAll) and toxic (MixTox) mixtures could point to antagonism between essential and toxic metals. None of the associations for selected metals/elements, two-way interactions or the joint mixture effect remained after adjusting for multiple comparisons. Therefore, the results must be interpreted with caution. Still, highlighted exposures herein should be considered important candidates for further studies of metal/element exposure and risk of CP in children, especially since very little knowledge exists to date on associations between toxicant and micronutrient levels during perinatal development and risk of CP.

The main source of Cu exposure in humans is food, and in some cases, drinking water (71). Cu is an essential trace mineral for many important enzymes and proteins in living organisms (72). It is important for foetal and child development, but excess levels can be toxic (17). There is evidence to support an adverse effect of Cu in human neurological disorders, such as Alzheimer, Huntington, and Menkes diseases (73). Possible mechanisms for Cu toxicity include its contribution in the formation of reactive oxygen species that modify the structure and/or function of essential biomolecules (72). Little is known about prenatal Cu and CP, and the present study is, to our knowledge, among the first to investigate and report this association. Only very few studies have investigated potential adverse effects of Cu on brain development, especially when it comes to psychomotor development (74, 75). Elevated airborne Cu exposure at school at ages 8 years and 12 years was associated with poorer motor performance and altered basal ganglia structure and function of the brain (76). Partly consistent with our findings, Amorós et al. (77) found inverse associations between Cu measured in maternal blood during the first trimester, and scores on neuropsychological development at one and 5 years. Also, in line with our findings where the risk of CP was higher among boys than compared to girls in relation to maternal Cu levels, Amorós et al. (77) found the associations to be strongest for boys at age 1 year. A stronger susceptibility for males to a range of toxicants is also found in several other studies (78). Further, the association between Cu and CP in the present study was strongest for lower concentrations of Pb, higher concentrations of Cd, and higher concentrations of Mn. Previous studies have shown that the toxicity of a toxicant can depend on the presence of other toxicants or elements (37, 79). Further, the effect of Cu was mainly found in children of less educated mothers, but not for children of highly educated mothers. Higher SES is associated with healthier lifestyle and living conditions (80), which might attenuate the adverse effects of increased Cu in the body. For example, studies have reported effect measure modification by SES in the relationship between Pb and adverse neurodevelopment, and it has been hypothesized that this might be due to differences in genetic susceptibilities, environmental enrichment, or stress (81).

Since preterm birth is an important predictor of CP (82), it was of special interest to see whether the effect estimates changed when children born before term were excluded from the analyses. For Cu, the estimate was slightly attenuated, indicating that the association for Cu might be stronger in preterm born children.

The maternal blood concentration of Cu in the present study (mean = 1,588 μg/L) was comparable to those in other studies, such as studies of pregnant women from Northern Norway (mean = 1,670 μg/L), Poland (mean = 1,694 μg/L), and Republic of Korea (mean = 1,650 μg/L) (83–85). Thus, the present study indicates that Cu blood levels during pregnancy might be associated with CP, even at concentration ranges commonly seen in populations.

For the trace element Mn measured in pregnancy, a positive association (increased risk) was identified with CP in the child. The main source of Mn exposure in humans is diet, and some industrial occupations (i.e., mining, welding and steel production) also represent a risk for increased Mn exposure by inhalation (86). As for Cu, excessive exposure can be neurotoxic and Mn is an established developmental neurotoxicant (31), although there is no clearly established mechanistic underpinning for its neurotoxicity. Mn could act through substituting calcium (Ca), and thus interfere with dopaminergic synaptic transmission, disruption of ATP synthesis in the mitochondria, and oxidization of dopamine, leading to increased intracellular oxidative stress (87, 88).

In adults, inhalation of Mn can lead to a condition called manganism, which is characterized by tremors, difficulties walking, and facial spasms. There is limited knowledge about early-life Mn exposure and neurological outcomes, but there is some evidence of adverse impact on cognition and behaviour (35, 89). Prenatal Mn concentrations in blood is associated with reduced birth weight and head and chest circumference (90). A South Korean study measured blood levels of Mn in pregnant women at term, and found associations with mental and psychomotor development at child age 6 months (91). A study on Italian adolescents found associations between increased soil Mn concentrations and impaired motor coordination and hand dexterity, and positive associations between blood and hair Mn concentrations and tremor intensity (22). Another study of children in Bangladesh found no associations between Mn in drinking water and Mn in blood and urine, and between Mn and motor function (23). However other neurodevelopmental outcomes (i.e., impaired cognitive function and academic achievement, internalizing and externalizing classroom behaviour) have been reported with elevated Mn exposure (92–94). The present study adds to the findings that increased Mn exposure is associated with adverse neurological effects in children. Contrary to some studies [i.e., (90)], the association in the present study was linear (positive), and not U- or inverted U-shaped. The maternal Mn concentrations in the present study (arithmetic mean = 11.5 μg/L, median = 9.8 μg/L, range: 1.9–163 μg/L) was similar or lower than reported in other studies general female United States population [median = 9.7 μg/L; Oulhote et al. (95)]; pregnant women during first trimester in the United States [median = 9.0 μg/L; Ashley-Martin et al. (96)]; and in mid-to-late-term pregnant Japanese women [median = 16.1 μg/L; Nakayama et al. (97)], and was within what is considered the normal range of 4 to 15 μg/L (87) [but higher in pregnant women (98)]. Thus, the present study indicates that elevated maternal levels of Mn in the second trimester can be associated with risk for CP, even at variation within normal concentration ranges.

The effect size of Cu and Mn found in the present study (OR for an increase in interquartile range = 1.40 and 1.20) is comparable to effect sizes of other reported risk factors for CP, such as low and high maternal age, low SES, and maternal hypertension during pregnancy (8).

Increased prenatal Hg exposure was associated with lowered odds of CP in the child. Much of the previous research indicates harmful effects of Hg on various human health outcomes, including adverse neurodevelopmental outcomes impaired motor function in children (25, 99). For example, cohort studies in the Faroe and Seychelles Islands found that increased prenatal MeHg exposure was related to lower scores on tests of motor function, coordination, fine motor skills, and motor speed in children (100–102). One of the most well-known examples is the MeHg poisoning in Minamata Bay in the 1950s (103). Hundreds of people died, after ingestion of MeHg-contaminated fish and shellfish from the Minamata Bay. Many people, especially children that were exposed to MeHg *in utero,* also displayed various adverse neurological effects, some similar to symptoms of CP. The Hg concentrations in Minamata were, however, much higher (i.e., hair concentrations of total Hg measured in 1960 was 15 times higher in Minamata than in the Kumamoto, a city located on the same island) than in the present study (104).

The inverse association between prenatal Hg exposure and odds of CP in the present study was unexcepted. This relationship also appeared to be driven by children of less educated mothers. Several studies report higher seafood intake in the higher SES strata, resulting in an elevated Hg prenatal exposure compared to the lower SES strata (105–107) since seafood is an important source of Hg (20). In a study of pregnant women from MoBa, Hg concentrations in blood were positively associated with total fish and seafood intake (45). Among the well-educated in the present study, the mothers of controls and CP cases ate similar amounts of seafood and Hg levels were approximately similar (Supplementary material 10). Among the less-educated, however, mothers of children with CP reported eating less seafood than mothers of controls, and they also appeared to have lower Hg blood concentrations than the mothers of controls (Supplementary material 10). In this study the analyses were adjusted for estimated maternal seafood intake. Even though it is possible that our results were biased by residual or unmeasured confounding, it could be speculated that if the Hg concentrations in blood is an even better marker of seafood intake than maternal, self-reported FFQ-based estimates of fish and seafood intake. If so, the seemingly lowered CP risk associated with increasing maternal Hg concentrations could in fact reflect increased intake of seafood and its beneficial nutrients for brain development (e.g., polyunsaturated fatty acids and iodine) (108).

The toxic metal mixture (MixTox) was inversely associated with risk of CP. This was mainly due to the inverse association between Hg and CP, reflected in the large negative weight for Hg (Supplementary material 13). There were no association between the essential element mixture (MixEssential) and CP or the total mixture and CP. Supplementary material 13 shows that the metal/element weights tend to go in opposite directions. In addition, some of the two-way interactions show that the effect of one metal or element is attenuated for certain concentration ranges of another metal or element (i.e., Cu and Pb). Nevertheless, the total mixture (MixAll) might have a stronger impact in other populations exposed to higher levels of toxic metals and/or with inadequate intake of essential elements or other micronutrients (e.g., folate), as they could have antagonistic effects.

This study has several strengths. First, it is among the first and largest studies of the association between prenatal exposure to toxic metals and essential elements, and CP diagnoses in children. Since only a relatively small percentage of children are diagnosed with CP (109), large studies are needed to identify a sufficient number of cases. The MoBa cohort was well suited for this purpose. Second, the scientific literature has called for investigation of chemical mixtures, as was done in the present study, which represent a more relevant exposure-scenario than assessing one or very few toxicants (37). By additionally assessing multiple two-way interactions between metals/elements, a more nuanced picture of the associations studied could be given. Third, our study was nested within a well-characterized prospective birth cohort with extensive questionnaire data that enabled us to obtain a wide range of relevant information on covariates.

Our study has some limitations. One limitation concerns self-selection bias into MoBa, resulting in larger proportion of older mothers with high education and healthier lifestyle than the general population (110). A second limitation is the lack of information on iron status. Iron is a main determinant for absorption of Mn and Cd in the body (79). Unfortunately, good measures of maternal iron levels were lacking in the present study so there is uncertainty regarding how well the levels of these metals reflect environmental exposure. Third, despite being one of the largest studies of its kind to date, the statistical power enabling identification of small to medium effect sized associations with intrauterine metal/element levels is probably restricted by the relatively low number of CP cases. Thus, future studies should strive to increase the case sample size.

## 5. Conclusion

When investigating the associations between gestational levels of 11 toxic metals and essential elements, within normal population ranges, Cu, Hg, and Mn were found to be associated with CP in children. Higher maternal levels of the essential elements Cu and Mn were associated with increased risk of CP in the child. While the total mixture effect was not found to be significant, counterintuitively, an inverse association between maternal Hg levels and risk of CP was also observed, and this association was mainly found in the lower SES strata. However, the inverse association reported herein should not be interpreted as a protective effect of Hg, but rather that Hg could be acting as a marker of seafood intake and nutrients that are beneficial for brain development. Disentangling adverse neurodevelopment of Hg or other contaminants originating from seafood intake and SES remains a great challenge within environmental epidemiology. The etiology of CP is a complex and multifactorial disease. Considerable research effort remains to elucidate the role of toxicants and micronutrients and their interactions during perinatal development in the etiology of CP in children, including increased attention to Cu and Mn. Consortium studies would be preferred, in order to produce larger case groups.

## Data availability statement

The data analyzed in this study is subject to the following licenses/restrictions: data from the Norwegian Mother, Father and Child Cohort Study (MoBa) and the Medical Birth Registry of Norway (MBRN) used in this study are managed by Norwegian Institute of Public Health and can be made available to researchers, provided approval from the Regional Committees for Medical and Health Research Ethics (REC), compliance with the EU General Data Protection Regulation (GDPR) and approval from the data owners. The consent given by the participants does not allow for storage of data on an individual level in repositories or journals. Researchers who want access to data sets for replication should apply through helsedata.no. Access to data sets requires approval from the Regional Committees for Medical and Health Research Ethics in Norway and an agreement with MoBa. Requests to access these datasets should be directed to helsedata.no, service@helsedata.no.

## Ethics statement

The studies involving human participants were reviewed and approved by The Regional Committees for Medical and Health Research Ethics. Written informed consent to participate in this study was provided by the participants’ legal guardian/next of kin.

## Author contributions

KW: writing—original draft, formal analysis, and methodology. AW: writing—second draft, review, formal analysis, and editing. PS, GA, TV, HK, CT, HM, and TS: writing—review and editing. GB and SE: writing—review and editing and methodology. HA: writing—review and editing and PI NeuroTox. GV: conceptualization, supervision, writing—review and editing, and methodology. All authors contributed to the article and approved the submitted version.

## Funding

This study was funded by the Research Council of Norway’s “NeuroTox” project (grant no. 267984/E50). The research is also conducted as part of the project Center for Global Health Inequalities Research (CHAIN) at the Norwegian University for Science and Technology (NTNU) financed by the Research Council of Norway (project no. 288638). The Norwegian Mother, Father, and Child Cohort Study (MoBa) is supported by the Norwegian Ministry of Health and Care Services and the Ministry of Education and Research, NIH/NINDS (grant nos. 1 UO1 NS 047537-01 and 2 UO1 NS 047537-06A1).

## Conflict of interest

The authors declare that the research was conducted in the absence of any commercial or financial relationships that could be construed as a potential conflict of interest.

## Publisher’s note

All claims expressed in this article are solely those of the authors and do not necessarily represent those of their affiliated organizations, or those of the publisher, the editors and the reviewers. Any product that may be evaluated in this article, or claim that may be made by its manufacturer, is not guaranteed or endorsed by the publisher.
