# Supplementary material for: Association between gestational levels of toxic metals and essential elements and cerebral palsy in children
Source: Front Neurol. 2023 Aug 17;14:1124943. doi: 10.3389/fneur.2023.1124943 (PMC10470125; doi:10.3389/fneur.2023.1124943)
Supplement: Supplementary file 1 [file Data_Sheet_1.docx]

## **Supplementary file 1. Detailed description of analytical procedures of biospecimens**

### Determination of metal and element concentrations in maternal blood

Maternal whole blood sampled in MoBa at approximately week 17 of pregnancy in 3 mL trace free sampling tubes and transported to the NIPH biobank for storage at – 80°C ^1^. Before analyses, the blood was thawed and 500 µl was aliquoted in matrix tubes (8 x 16) and re-frozen. The samples were shipped frozen on dry ice to the respective analytical laboratories.

The majority of the maternal blood samples (n=105) were analysed by ALS Laboratory group, at the ALS laboratory in Luleå, Sweden in 2015-2016. In addition to the maternal blood samples, five reference samples consisting of standard reference material (Seronorm Urine L-1: Sero AS, Billingstad, Norway) were randomly placed within the sample batches, blinded to the analyst.

The analytical method at ALS laboratory group is accredited according to the standard EN ISO 17294-2:2016. The analyses included the following toxic or non-essential metals/elements: Lead (Pb), total Mercury (Hg), Cadmium (Cd), total Arsenic (As), and Cesium (Cs), and as well as the following essential metals/elements: Cobolt (Co), Magnesium (Mg), Copper (Cu), Selenium (Se), Manganese (Mg), and Zink (Zn). The method have been described previously ^2,3^. Briefly, it included microwave-assisted sample decomposition followed by inductively coupled plasma-sector field mass spectrometry (ICP-SFMS). Sample blanks (200 µl of high purity water), control samples (Seronorm Urine L-2: Sero AS, Billingstad, Norway) and calibration standards were subjected to the same handling as samples, including the addition of internal standards. The concentration of metals/elements in blood and blind samples, blanks and laboratory controls were calculated based on calibration curves. Limit of quantifications (LOQ) was calculated as ten times the standard deviation of the blank samples, whereas limit of detection (LOD) was approximately one third of the LOQ. The LOD and LOQ for the various elements are presented in Table S1. All reported concentrations in this study were above LOQ, except for As, Hg, Cd and Pb where concentrations above LOD were reported.

Results of maternal whole blood concentrations of metals and essential elements for some of our participants (n=105) were provided by The Norwegian Environmental Biobank, a sub-study of MoBa. These samples were analysed 2015-2016 at the Department of Occupational and Environmental Medicine at Lund University, Sweden, using comparable analytical methods as the ALS Laboratory group, as described in Caspersen et al. (2019)^4^, however, these analyses did not include Mg or Cs. All metals and elements, except Total Hg, were analysed using ICP-MS (iCap Q Thermo Fisher Scientific, Bremen, GmbH) according to previous method descriptions ^4,5^. Total Hg was determined by cold vapor fluorescence spectrometry after acid-digestion of samples ^4,6^. The LOD and LOQ of the metals/elements measured by Lund University is given in Caspersen et al. ^4^

Certified reference material (Seronorm Trace Elements whole blood L-1, SERO AS, Billingstad, Norway) were used to ensure the analytical accuracy at both laboratories. The intra-laboratory coefficients of variation were within acceptable ranges for ALS Laboratory group (Table S1) and Lund University (see Caspersen et al. 2019 ^4^).

## **Supplementary file 2. Description of elastic net and the false discovery rate method used in the present study, based on an algorithm by Ahmed et al^7^.**

### Elastic net (Zou and Hastie 2005^8^):

The ordinary least squares (OLS) estimator is unbiased, but can have a large variance, especially if variables are highly correlated or there are many variables in the model. Regularization methods are a way of reducing this large variance, at the expense of introducing some bias in the model. Examples include ridge regression, where coefficients are shrunk towards zero, but all variables are retained in the model. In lasso regression, the penalty/bias applied lead to shrinkage of coefficients towards zero, and can sometimes result in coefficients equal to zero, thus performing variable selection. Lasso works best if the distinction between good and bad predictors is clear. If there is strong correlation between two variables, lasso tend to pick one. To overcome limitations of the lasso, elastic net regression combines the penalties of ridge and lasso, making it able to select important variables, despite their being strongly correlated.

In the present study, alpha was set to 0.9. Lambda was selected using cross validation, and we selected the lambda value that minimized the mean squared error.

### False discovery rate (FDR) method:

First, 1000 new datasets were made by permuting the outcome variable. Permutation is supposed to break up any possible association between exposure and outcome variables. In each of these 1000 datasets, random sampling with replacement was done 50 times, and in each of these 50 datasets, 20 multiple imputed datasets were made. Elastic net was run in all the multiple imputed datasets. This means that the stability selection approach described above was repeated 1000 times in datasets where the null hypotheses were true (assumingly), due to the permutation. P-values were then calculated by summing up how many times the selection probability in the permuted datasets was equal to or greater than the selection probability based on original data, and then dividing this sum by the number of permuted datasets. This p-values were ranked from smallest to largest and compared to the Benjamini and Hochberg false discovery rate threshold ^9^, given by:

P_FDR_ = $\frac{\alpha*k}{n}$ ,

where α is the desired significance level at which to control the fdr; *k* is the number in the rank; n is the number of tests done.

When analyzing interaction terms, 10.000 permuted datasets were used, and alpha was set to 0.1.

**Supplementary file 3. Limit of detection (LOD) and quantification (LOQ), and quality control data for the analyses of metals and elements in whole blood at ALS Laboratory group. Concentrations are given in microgram per milliliter (μg/mL)**

| ALS Laboratory group – MoBa NeuroTox project | | | | | | | |
| --- | --- | --- | --- | --- | --- | --- | --- |
|  |  |  | Reference samples ^a,b^ |  |  |  |  |
| Metal/ element | ~LOD | LOQ | Measured Mean±SD (RSD) | Target Mean±SD | Mean deviation from target | n<LOD(%)/  n<LOQ(%) | n missing (%) |
| As | 0.3 | 1.0 | 2.7±0.5 (19%) | 2.4±0.5 | 12% | 36(2.5)/351  (28.6) | - |
| Pb | 0.15 | 0.5 | 10.1±0.7 (7%) | 10.2±2.1 | 0.9% | 0 | 0 |
| Cd | 0.02 | 0.05 | 0.35±0.03 (8%) | 0.36 ± 0.02 | 2.2% | 29(2.4)/66  (5.4) | 0 |
| Cs | 0.02 | 0.05 | 2.52±0.11 (4%) | 2.5±0.03 | 0.8% | 0 | 105(8.6) |
| Hg | 0.07 | 0.2 | 1.8±0.1 (5%) | 1.5±0.3 | 21% | 0 | 0 |
| Co | 0.02 | 0.05 | 0.21±0.03 (14%) | 0.16±0.03 | 33% | 33()/45  (3.7) | - |
| Cu | 0.3 | 1.0 | 656±49.7(8%) | 680±140 | 3.5% | 0 | 0 |
| Mn | 0.15 | 0.5 | 22.4± 5.3 (24%) | 20.7±4.2 | 8.2% | 0 | 0 |
| Se | 1.7 | 5.0 | 60.9±7.4 (12%) | 59.0±12.0 | 3.2% | 0 | 0 |
| Zn | 3.3 | 10 | 4056±147.7 (4%) | 4400±200 | 7.8% | 0 | 0 |
| Mg | 0.07 | 0.2 | 14.6±1.52 (10%) | 16.2±1,6 | 9.9% | 0 | 105(8.6) |
| ^a^ n = 5 blind samples, ^b^ Seronorm. Abbreviations: Arsenic (As); cadmium (Cd); cesium (Cs); cobalt (Co); copper (Cu); lead (Pb); limit of detection (LOD); limit of quantification (LOQ); magnesium (Mg); manganese (Mn); mercury (Hg); relative standard deviation (RSD); selenium (Se); standard deviation (SD); zinc (Zn). | | | | | | | |

## **Supplementary file 4. Unadjusted metal/element concentrations (μg/L or mg/L) in a nested case–control study of cerebral palsy in The Norwegian Mother, Father and Child Cohort Study (MoBa), 2002–2006.**

|  | **Controls** | **CP cases** | **Total** |
| --- | --- | --- | --- |
|  | **N=1082**  **Mean(SD), min, 10%, 50%, 90%, max** | **N=144**  **Mean(SD), min, 10%, 50%, 90%, max** | **N=1226**  **Mean(SD), min, 10%, 50%, 90%, max** |
| **Hg** | 1.6(1.0) 0.1 0.6 1.4 2.9 10.9 | 1.5(0.9) 0.1 0.5 1.3 2.8 4.5 | 1.6(1.0) 0.1 0.6 1.4 2.9 10.9 |
| **As** | 2.4(3.1) 0.1 0.6 1.6 4.7 51.6 | 2.5(2.8) 0.2 0.9 1.7 4.5 18.1 | 2.4(3.0) 0.1 0.6 1.6 4.7 51.6 |
| **MISSING** | 12 | 0 | 12 |
| **Cd** | 0.3(0.3) 0.0 0.1 0.2 0.5 3.1 | 0.2(0.2) 0.0 0.1 0.2 0.3 2.2 | 0.3(0.3) 0.0 0.1 0.2 0.5 3.1 |
| **MISSING** | 22 | 0 | 22 |
| **Pb** | 9.3(5.0) 1.9 5.2 8.4 13.9 78.6 | 9.0(7.0) 0.9 4.5 8.0 13.4 79.0 | 9.3(5.3) 0.9 5.2 8.3 13.8 79.0 |
| **Cs** | 2.4(0.9) 0.9 1.5 2.2 3.3 8.4 | 2.2(0.8) 0.8 1.4 2.2 3.2 5.2 | 2.3(0.9) 0.8 1.5 2.2 3.3 8.4 |
| **MISSING** | 103 | 2 | 105 |
| **Mn** | 11.1(8.6) 3.3 6.6 9.8 15.2 158.0 | 13.0(12.5) 1.8 6.5 10.0 18.8 102.0 | 11.4(9.1) 1.8 6.6 9.8 15.4 158.0 |
| **Se** | 96.9(21.3) 48.1 75.1 94.1 122.0 319.0 | 98.1(20.0) 60.6 74.0 96.2 130.0 154.0 | 97.1(21.1) 48.1 75.0 94.3 122.8 319.0 |
| **Co** | 0.3(0.9) 0.0 0.1 0.2 0.4 26.8 | 0.2(0.2) 0.0 0.1 0.2 0.3 1.4 | 0.2(0.8) 0.0 0.1 0.2 0.4 26.8 |
| **MISSING** | 32 | 1 | 33 |
| **Mg** | 29.6(3.4) 17.9 25.4 29.5 33.9 44.8 | 30.4(4.1) 17.9 24.8 30.4 35.8 41.0 | 29.7(3.5) 17.9 25.3 29.6 34.2 44.8 |
| **MISSING** | 103 | 2 | 105 |
| **Cu** | 1505(241) 712 1230 1480 1810 3210 | 1636(283) 948 1290 1625 1990 3100 | 1520(250) 712 1230 1490 1840 3210 |
| **Zn** | 5229(1007) 1650 3960 5185 6490 9500 | 5222(906) 1440 4080 5255 6300 7110 | 5228(995) 1440 3980 5200 6460 9500 |
| Note: Mg values are in mg/L, all others are in μg/L. Abbreviations: Arsenic (As); cadmium (Cd); cerebral palsy (CP); cesium (Cs); cobalt (Co); copper (Cu); lead (Pb); magnesium (Mg); manganese (Mn); mercury (Hg); selenium (Se); zinc (Zn). | | | |

## **Supplementary file 5. Main effects. Results of stability selection using elastic net regression, calculated p-values and Benjamini and Hochberg false discovery rate threshold (p_FDR_) in a nested case–control study of cerebral palsy in The Norwegian Mother, Father and Child Cohort Study (MoBa), 2002–2006.**

|  | **Mean selection probability** | **Calculated p-value** | **p_FDR_** |
| --- | --- | --- | --- |
| **logCu** | 0.88 | 0.018 | 0.005 |
| **logHg** | 0.87 | 0.019 | 0.009 |
| **logMn** | 0.79 | 0.055 | 0.014 |
| **logZn** | 0.70 | 0.086 | 0.018 |
| **logCs** | 0.63 | 0.190 | 0.032 |
| **logMg** | 0.62 | 0.150 | 0.023 |
| **logSe** | 0.58 | 0.180 | 0.027 |
| **logCo** | 0.54 | 0.270 | 0.041 |
| **logPb** | 0.53 | 0.250 | 0.036 |
| **logAs** | 0.41 | 0.430 | 0.045 |
| **logCd** | 0.40 | 0.500 | 0.050 |
| Note: Mean selection probabilities based on 2.000 elastic net regression runs (once in each of 20 multiple imputed datasets in each of 100 randomly sampled datasets with replacement). Calculated p-values Supplementaryly based on 240.000 elastic net runs (once in each of 10 multiple imputed datasets in each of 20 randomly sampled datasets with replacements in each of 1200 permuted datasets). N=1226. Abbreviations: Arsenic (As); cadmium (Cd); cesium (Cs); cobalt (Co); copper (Cu); lead (Pb); magnesium (Mg); manganese (Mn); mercury (Hg); selenium (Se); zinc (Zn). | | | |

## **Supplementary file 6. Main analyses, main effects. Logistic regression odds ratio estimates for metals from single exposure models, and from logistic regression models including all selected exposures** **in a nested case–control study of cerebral palsy in The Norwegian Mother, Father and Child Cohort Study (MoBa), 2002–2006.**

|  | **Single exposure** | | | **Selected exposures** | **LR-test,**  **linear vs splines** | **Single exposure.**  **Corrected for**  **multiple comparisons*** | |
| --- | --- | --- | --- | --- | --- | --- | --- |
|  | OR | 95% CI | | OR | p-value | 99.6% CI | |
| **logAs** | 0.95 | 0.73 | 1.20 |  |  | 0.64 | 1.40 |
| **logHg** | 0.69 | 0.51 | 0.92 | 0.68 | 0.31 | 0.44 | 1.05 |
| **logSe** | 1.10 | 0.83 | 1.40 |  |  | 0.74 | 1.52 |
| **logCd** | 0.97 | 0.81 | 1.20 |  |  | 0.74 | 1.30 |
| **logPb** | 0.95 | 0.73 | 1.20 |  |  | 0.64 | 1.40 |
| **logMn** | 1.30 | 1.00 | 1.50 | 1.20 | 0.31 | 0.91 | 1.70 |
| **logCu** | 1.50 | 1.10 | 2.00 | 1.40 | 0.18 | 0.99 | 2.20 |
| **logCs** | 0.84 | 0.64 | 1.10 |  |  | 0.55 | 1.26 |
| **logCo** | 0.94 | 0.74 | 1.20 |  |  | 0.65 | 1.32 |
| **logZn** | 0.88 | 0.69 | 1.10 |  |  | 0.62 | 1.30 |
| **logMg** | 1.20 | 0.90 | 1.50 |  |  | 0.81 | 1.73 |
| **MixAll.** | 1.00 | 0.67 | 1.50 |  |  | 0.57 | 1.80 |
| **MixEssential.** | 1.30 | 0.93 | 2.50 |  |  | 0.81 | 2.20 |
| **MixToxic.** | 0.77 | 0.56 | 1.00 |  |  | 0.49 | 1.20 |
| Note: Odds ratios. Per interquartile range increase in exposure. Models fitted in 20 multiple imputed datasets, and estimates combined using Rubin’s rules. *MixAll contains all 11 metals; MixEssential contains Se, Mn, Cu, Co, Zn, and Mg; MixToxic contains As, Hg, Cd, Pb, and Cs. All models adjusted for maternal age at delivery, maternal seafood intake during pregnancy, maternal smoking during pregnancy, parity, maternal education, birth year, and child’s sex.*) Sidak-corrected. Abbreviations: Arsenic (As); cadmium (Cd); cesium (Cs); cobalt (Co); copper (Cu); lead (Pb); likelihood ratio (LR); magnesium (Mg); manganese (Mn); mercury (Hg); selenium (Se); zinc (Zn). | | | | | | | |

## **Supplementary file 7. Logistic regression odds ratios of sensitivity analyses including the selected exposures in a nested case–control study of cerebral palsy in The Norwegian Mother, Father and Child Cohort Study (MoBa), 2002–2006.**

|  | **Main**  **N=1226**  **OR (95% CI)** | **No premature**  **N=1138**  **OR (95% CI)** | **Folate below median**  **N=613**  **OR (95% CI)** | **Folate above median**  **N=613**  **OR (95% CI)** | **No smoking**  **N=1069**  **OR (95% CI)** | **No SGA**  **N=1199**  **OR (95% CI)** | **No winsorizing**  **N=1226**  **OR (95% CI)** | **Complete cases**  **N=1002**  **OR (95% CI)** |
| --- | --- | --- | --- | --- | --- | --- | --- | --- |
| **logCu** | 1.40 | 1.20 | 1.00 | 1.80 | 1.50 | 1.40 | 1.40 | 1.40 |
| **logHg** | 0.68 | 0.66 | 0.75 | 0.60 | 0.70 | 0.66 | 0.67 | 0.68 |
| **logMn** | 1.20 | 1.20 | 1.20 | 1.30 | 1.20 | 1.20 | 1.20 | 1.10 |
| **MixAll** | 1.00 (0.67, 1,50) | 0.86 (0.54, 1.40) | 0.94 (0.49, 1.80) | 1.10 (0.57, 2.00) | 1.10 (0.74, 1.70) | 1.10 (0.69, 1.60) | 1.00 (0.67, 1.50) | 1.10 (0.65, 1.80) |
| **MixEssential** | 1.30 (0.93, 2,50) | 1.10 (0.79, 1.60) | 1.20 (0.70, 2.00) | 1.40 (0.85, 2.30) | 1.40 (1.00, 2.00) | 1.30 (0.94, 2.60) | 1.30 (0.93, 2.50) | 1.40 (0.95, 2.00) |
| **MixToxic** | 0.77 (0.56, 1.00) | 0.72 (0.50, 1.00) | 0.86 (0.52, 1.40) | 0.69 (0.44, 1.10) | 0.78 (0.57, 1.10) | 0.79 (0.57, 1.10) | 0.77 (0.56, 1.00) | 0.76 (0.53, 1.10) |
| Note: Odds ratios. Per interquartile range increase in exposure. Models fitted in 20 multiple imputed datasets, and estimates combined using Rubin’s rules. All models adjusted for maternal age at delivery, maternal seafood intake during pregnancy, maternal smoking during pregnancy, parity, maternal education, birth year, and child’s sex. Abbreviations: Copper (Cu); manganese (Mn); mercury (Hg). | | | | | | | | |

## **Supplementary file 8. Two-way interactions. Results of stability selection using elastic net regression, calculated p-values and Benjamini and Hochberg false discovery rate threshold (p_FDR_) in a nested case–control study of cerebral palsy in The Norwegian Mother, Father and Child Cohort Study (MoBa), 2002–2006.**

|  | **Mean selection probability** | **Calculated p-value** | **BH threshold** |
| --- | --- | --- | --- |
| logCu:logPb | 0.890 | 0.017 | 0.001 |
| logCd:logCu | 0.819 | 0.065 | 0.006 |
| Maternal_edu:logCu | 0.793 | 0.059 | 0.004 |
| logHg:logMg | 0.787 | 0.025 | 0.003 |
| Maternal_edu:logHg | 0.743 | 0.061 | 0.005 |
| logCd:logPb | 0.733 | 0.088 | 0.009 |
| KJONN:logCu | 0.732 | 0.090 | 0.010 |
| logCu:logMn | 0.722 | 0.096 | 0.012 |
| logCo:logHg | 0.697 | 0.084 | 0.008 |
| logAs:logCd | 0.623 | 0.131 | 0.013 |
| logCo:logCu | 0.610 | 0.189 | 0.016 |
| logCu:logSe | 0.609 | 0.180 | 0.014 |
| KJONN:logCo | 0.574 | 0.248 | 0.021 |
| logCd:logCs | 0.542 | 0.242 | 0.018 |
| logMn:logPb | 0.524 | 0.245 | 0.019 |
| logAs:logHg | 0.510 | 0.299 | 0.023 |
| logCd:logMg | 0.501 | 0.199 | 0.017 |
| logCs:logMn | 0.492 | 0.279 | 0.022 |
| Maternal_edu:logCo | 0.482 | 0.326 | 0.025 |
| Maternal_edu:logSe | 0.474 | 0.334 | 0.027 |
| logCo:logMn | 0.469 | 0.336 | 0.030 |
| Maternal_edu:logMn | 0.462 | 0.373 | 0.036 |
| logCs:logPb | 0.455 | 0.331 | 0.026 |
| logCo:logZn | 0.454 | 0.344 | 0.032 |
| logMn:logSe | 0.452 | 0.336 | 0.031 |
| logMn:logMg | 0.446 | 0.353 | 0.034 |
| logZn:logMg | 0.438 | 0.407 | 0.038 |
| logMn:logZn | 0.428 | 0.335 | 0.029 |
| logCo:logCs | 0.422 | 0.363 | 0.035 |
| KJONN:logCs | 0.418 | 0.411 | 0.040 |
| Maternal_edu:logCs | 0.416 | 0.432 | 0.044 |
| KJONN:logPb | 0.412 | 0.473 | 0.052 |
| logCu:logHg | 0.388 | 0.419 | 0.042 |
| KJONN:logHg | 0.381 | 0.452 | 0.048 |
| logHg:logMn | 0.380 | 0.410 | 0.039 |
| logPb:logSe | 0.378 | 0.447 | 0.047 |
| logAs:logZn | 0.371 | 0.420 | 0.043 |
| KJONN:logCd | 0.364 | 0.547 | 0.058 |
| KJONN:logAs | 0.364 | 0.548 | 0.060 |
| logHg:logPb | 0.354 | 0.467 | 0.051 |
| KJONN:logMn | 0.354 | 0.598 | 0.065 |
| KJONN:logMg | 0.351 | 0.501 | 0.053 |
| logCs:logSe | 0.348 | 0.538 | 0.057 |
| logHg:logSe | 0.342 | 0.533 | 0.056 |
| logCs:logZn | 0.339 | 0.513 | 0.055 |
| logCu:logMg | 0.338 | 0.588 | 0.064 |
| logSe:logZn | 0.336 | 0.443 | 0.045 |
| Maternal_edu:logMg | 0.316 | 0.554 | 0.062 |
| logAs:logMg | 0.313 | 0.456 | 0.049 |
| logCd:logSe | 0.306 | 0.632 | 0.068 |
| KJONN:logSe | 0.306 | 0.749 | 0.077 |
| logSe:logMg | 0.304 | 0.552 | 0.061 |
| logAs:logCo | 0.302 | 0.611 | 0.066 |
| logCd:logMn | 0.293 | 0.635 | 0.069 |
| Maternal_edu:logPb | 0.293 | 0.747 | 0.075 |
| logCo:logCd | 0.292 | 0.768 | 0.081 |
| logCs:logCu | 0.288 | 0.713 | 0.073 |
| logPb:logMg | 0.282 | 0.673 | 0.070 |
| KJONN:logZn | 0.271 | 0.756 | 0.079 |
| logCd:logZn | 0.268 | 0.674 | 0.071 |
| logCo:logSe | 0.267 | 0.797 | 0.088 |
| logCo:logPb | 0.264 | 0.778 | 0.083 |
| logCs:logMg | 0.259 | 0.720 | 0.074 |
| logCo:logMg | 0.247 | 0.751 | 0.078 |
| logAs:logCu | 0.244 | 0.781 | 0.084 |
| logAs:logCs | 0.243 | 0.796 | 0.087 |
| logAs:logSe | 0.236 | 0.826 | 0.090 |
| logAs:logPb | 0.235 | 0.775 | 0.082 |
| Maternal_edu:logCd | 0.228 | 0.885 | 0.096 |
| Maternal_edu:logZn | 0.224 | 0.852 | 0.094 |
| logCs:logHg | 0.212 | 0.847 | 0.092 |
| logAs:logMn | 0.209 | 0.793 | 0.086 |
| logCd:logHg | 0.208 | 0.839 | 0.091 |
| logPb:logZn | 0.198 | 0.854 | 0.095 |
| logCu:logZn | 0.188 | 0.901 | 0.097 |
| Maternal_edu:logAs | 0.183 | 0.929 | 0.100 |
| logHg:logZn | 0.148 | 0.927 | 0.099 |
| Note: Mean selection probabilities based on 4.000 elastic net regression runs (once in each of 20 multiple imputed datasets in each of 200 randomly sampled datasets with replacement). Calculated p-values Supplementaryly based on 1.000.000 elastic net runs (once in each of 5 multiple imputed datasets in each of 20 randomly sampled datasets with replacements in each of 10000 permuted datasets). N=1226. Abbreviations: Arsenic (As); cadmium (Cd); cesium (Cs); cobalt (Co); copper (Cu); lead (Pb); magnesium (Mg); manganese (Mn); mercury (Hg); selenium (Se); zinc (Zn). | | | |

## **Supplementary file 9. Two-way interactions, ORs for interactions with p<0.1, with corresponding main effect terms in a nested case–control study of cerebral palsy in The Norwegian Mother, Father and Child Cohort Study (MoBa), 2002–2006.**

| **Main and interaction term,**  **standardized** | **OR** |
| --- | --- |
| Cu | 0.251 |
| Pb | -0.008 |
| CuXPb | -0.190 |
| Hg | -0.274 |
| Mg | 0.200 |
| HgXMg | 0.197 |
| M_Edu | -0.282 |
| Cu | 0.546 |
| CuXM_Edu | -0.423 |
| M_Edu | -0.268 |
| Hg | -0.474 |
| HgXM_Edu | 0.350 |
| Cu | 0.305 |
| Cd | -0.070 |
| CuXCd | 0.228 |
| Hg | -0.260 |
| Co | -0.095 |
| HgXCo | -0.185 |
| Cd | -0.044 |
| Pb | -0.051 |
| CdXPb | -0.161 |
| Sex | 0.515 |
| Cu | 0.435 |
| CuXSex | -0.418 |
| Cu | 0.245 |
| Mn | 0.088 |
| CuXMn | 0.148 |
| Abbreviations: Arsenic (As); cadmium (Cd); cesium (Cs); cobalt (Co); copper (Cu); lead (Pb); magnesium (Mg); manganese (Mn); mercury (Hg); selenium (Se); zinc (Zn). | |

## **Supplementary file 10. Comparison of Hg concentrations and seafood intake in mothers with lower and higher education** **in a nested case–control study of cerebral palsy in The Norwegian Mother, Father and Child Cohort Study (MoBa), 2002–2006.**

|  | mean.hg.CP0 | mean.hg.CP1 | median.hg.CP0 | median.hg.CP1 | seafood.CP0 | seafood.CP1 | hg.OR |
| --- | --- | --- | --- | --- | --- | --- | --- |
| Lower edu | 1.045 | 0.754 | 1.063 | 0.722 | 36.401 | 27.076 | 0.366 |
| Higher edu | 1.204 | 1.103 | 1.243 | 1.252 | 36.620 | 36.048 | 0.784 |

## **Supplementary file 11**


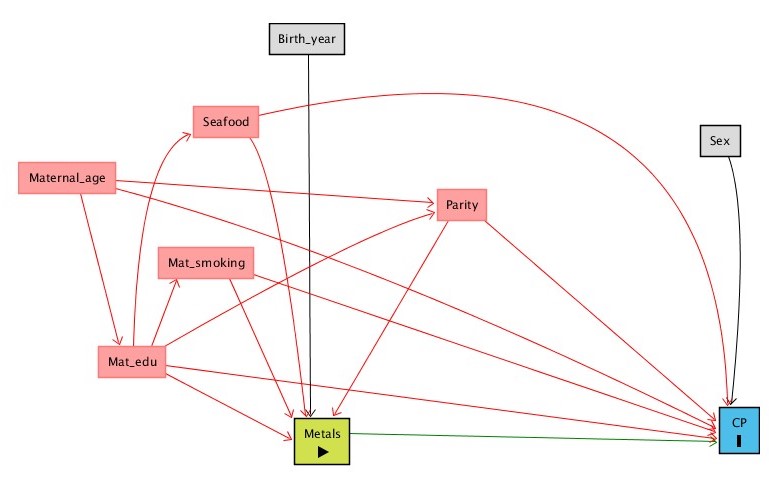


**Directed acyclic graph (DAG) in a nested case–control study of cerebral palsy in The Norwegian Mother, Father and Child Cohort Study (MoBa), 2002–2006**.

## **Supplementary file 12**


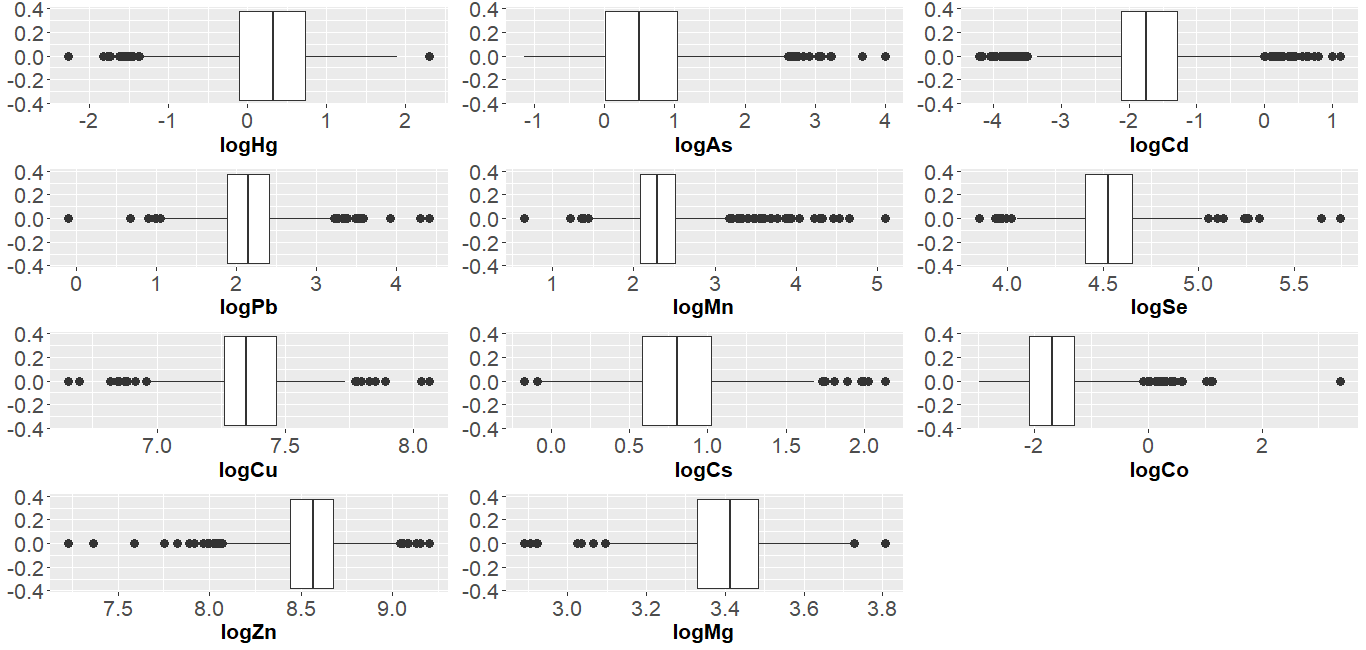


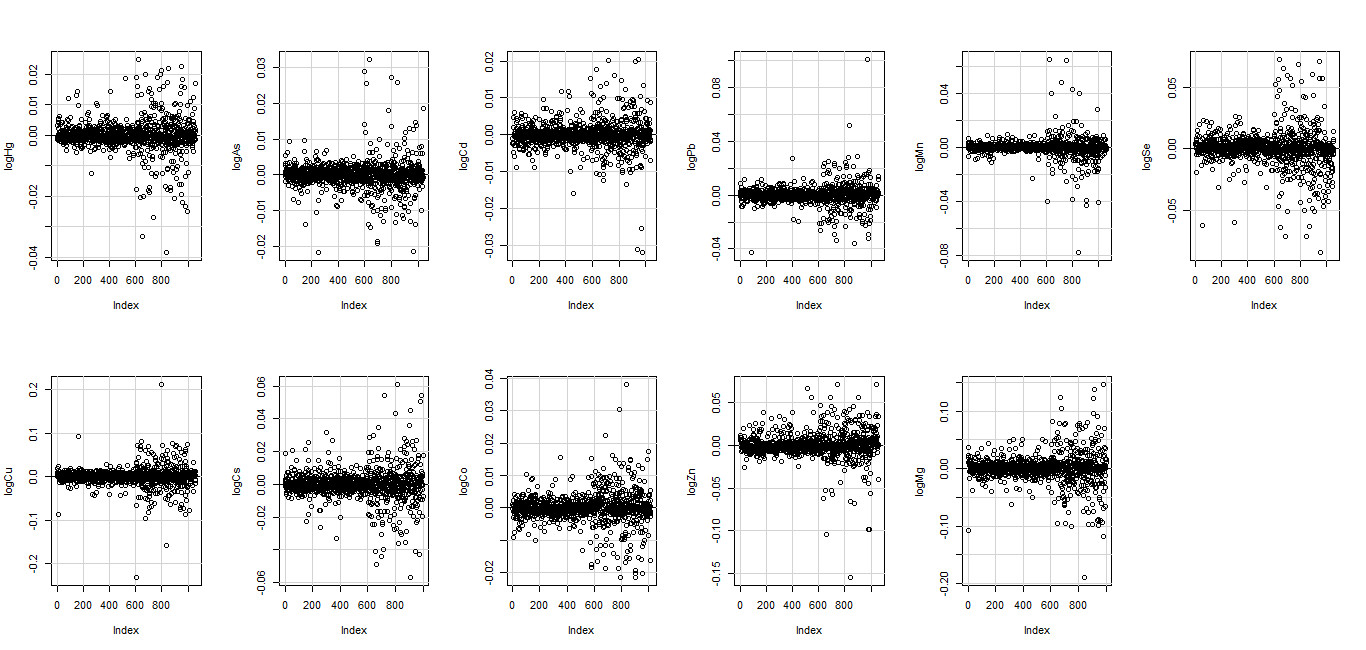


**Box plots in a nested case–control study of cerebral palsy in The Norwegian Mother, Father and Child Cohort Study (MoBa), 2002–2006.**

Upper: Box plots of unadjusted metal/element concentrations. Lower: dfbetas for all unadjusted metal/element concentrations, where the dfbeta value is considered influential if it exceeds ±2/sqrt(n), or, in this case ± 0.057. Abbreviations: Arsenic (As); cadmium (Cd); cesium (Cs); cobalt (Co); copper (Cu); lead (Pb); magnesium (Mg); manganese (Mn); mercury (Hg); selenium (Se); zinc (Zn).

## **Supplementary file 13**


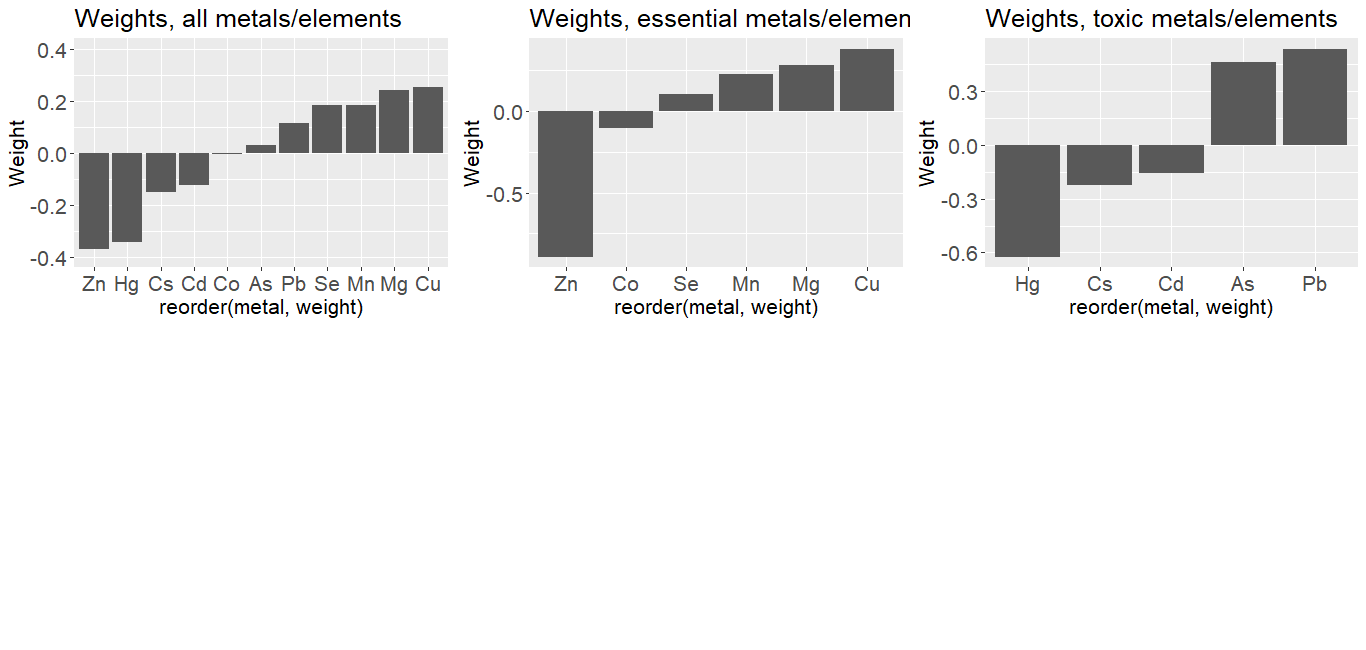


**Weights from the quantile g-computation analyses (average weights across 20 imputed datasets)** **in a nested case–control study of cerebral palsy in The Norwegian Mother, Father and Child Cohort Study (MoBa), 2002–2006**.

Models are adjusted for the minimal adjustment set of covariates. N=1226. Abbreviations: Arsenic (As); cadmium (Cd); cesium (Cs); cobalt (Co); copper (Cu); lead (Pb); magnesium (Mg); manganese (Mn); mercury (Hg); selenium (Se); zinc (Zn).

## **Supplementary file 14**


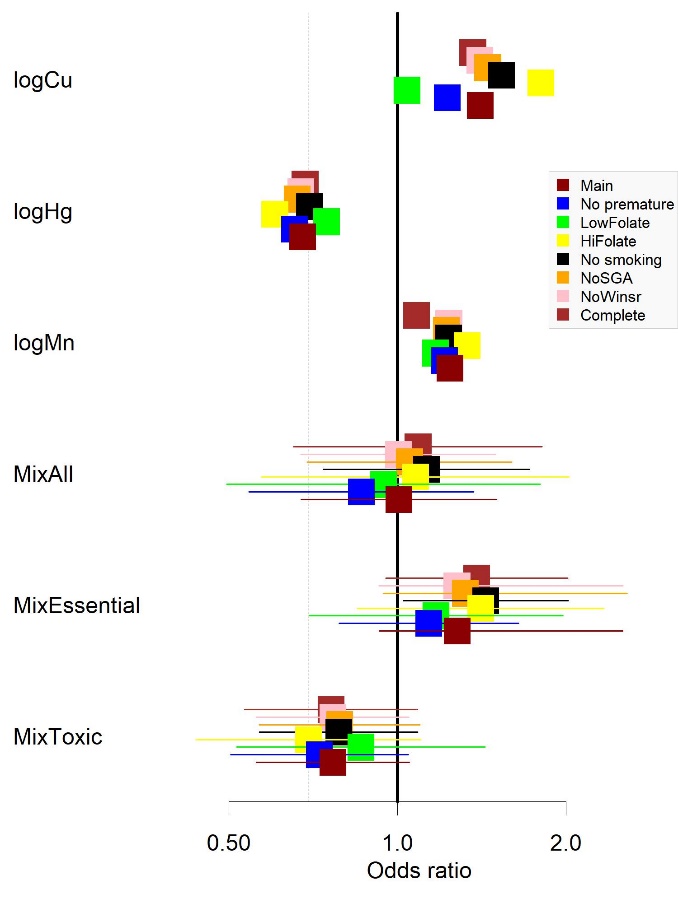


**Sensitivity analyses in a nested case–control study of cerebral palsy in The Norwegian Mother, Father and Child Cohort Study (MoBa), 2002–2006**.

Logistic regression ORs for selected metals. Single metal/element: per interquartile range increase in exposure; mix: per quartile increase in mix. Based on 20 multiple imputed datasets. Models adjusted for minimal adjustment set of covariates. N_Original_=1226, N_NoPremature_=1138, N_LowFolate_=613, N_HighFolate_=613, N_NoSmoking_=1069, N_Complete_=1002.

**References**

1 Paltiel, L. *et al.* The biobank of the Norwegian mother and child cohort study - Present status. *Norsk Epidemiologi* **24**, 29-35 (2014).

2 Rodushkin, I. Determination of trace and ultratrace elements inbody fluids using sector-field ICP-MS. (2004).

3 Rodushkin, I., Ödman, F., Olofsson, R. & Axelsson, M. D. Determination of 60 elements in whole blood by sector field inductively coupled plasma mass spectrometry. *Journal of Analytical Atomic Spectrometry* **15**, 937-944, doi:10.1039/B003561K (2000).

4 Caspersen, I. H. *et al.* Patterns and dietary determinants of essential and toxic elements in blood measured in mid-pregnancy: The Norwegian Environmental Biobank. *Sci. Total Environ.* **671**, 299-308, doi:<https://doi.org/10.1016/j.scitotenv.2019.03.291> (2019).

5 Barany, E., Bergdahl, I. A., SchÜTz, A., Skerfving, S. & Oskarsson, A. Inductively Coupled Plasma Mass Spectrometry for Direct Multi-element Analysis of Diluted Human Blood and Serum. *Journal of Analytical Atomic Spectrometry* **12**, 1005-1009, doi:10.1039/A700904F (1997).

6 Sandborgh-Englund, G., Elinder, C. G., Langworth, S., Schutz, A. & Ekstrand, J. Mercury in biological fluids after amalgam removal. *J. Dent. Res.* **77**, 615-624, doi:10.1177/00220345980770041501 (1998).

7 Ahmed, I., Hartikainen, A.-L., Järvelin, M.-R. & Richardson, S. False Discovery Rate Estimation for Stability Selection: Application to Genome-Wide Association Studies. *Statistical Applications in Genetics and Molecular Biology* **10**, doi:<https://doi.org/10.2202/1544-6115.1663> (2011).

8 Zou, H. & Hastie, T. Regularization and variable selection via the elastic net. *Journal of the Royal Statistical Society: Series B (Statistical Methodology)* **67**, 301-320, doi:<https://doi.org/10.1111/j.1467-9868.2005.00503.x> (2005).

9 Benjamini, Y. & Hochberg, Y. Controlling The False Discovery Rate - A Practical And Powerful Approach To Multiple Testing. *J. Royal Statist. Soc., Series B* **57**, 289-300, doi:10.2307/2346101 (1995).
